# Residual cancer burden after neoadjuvant chemotherapy and long-term survival outcomes in breast cancer: a multicentre pooled analysis of 5161 patients

**DOI:** 10.1016/S1470-2045(21)00589-1

**Published:** 2021-12-11

**Authors:** Christina Yau, Marie Osdoit, Marieke van der Noordaa, Sonal Shad, Jane Wei, Diane de Croze, Anne-Sophie Hamy, Marick Laé, Fabien Reyal, Gabe S Sonke, Tessa G Steenbruggen, Maartje van Seijen, Jelle Wesseling, Miguel Martín, Maria del Monte-Millán, Sara López-Tarruella, I-SPY 2 Trial Consortium, Judy C Boughey, Matthew P Goetz, Tanya Hoskin, Rebekah Gould, Vicente Valero, Stephen B Edge, Jean E Abraham, John M S Bartlett, Carlos Caldas, Janet Dunn, Helena Earl, Larry Hayward, Louise Hiller, Elena Provenzano, Stephen-John Sammut, Jeremy S Thomas, David Cameron, Ashley Graham, Peter Hall, Lorna Mackintosh, Fang Fan, Andrew K Godwin, Kelsey Schwensen, Priyanka Sharma, Angela M DeMichele, Kimberly Cole, Lajos Pusztai, Mi-Ok Kim, Laura J van ‘t Veer, Laura J Esserman, W Fraser Symmans

**Affiliations:** Department of Surgery (C Yau PhD, M Osdoit MD, M van der Noordaa MD, S Shad BS, J Wei BS, Prof L J Esserman MD), Department of Epidemiology and Biostatistics (Prof M-O Kim PhD), and Department of Laboratory Medicine (Prof L J van ‘t Veer PhD), University of California, San Francisco, San Francisco, CA, USA; Department of Surgery (M Osdoit, Prof F Reyal MD), Department of Tumor Biology (D de Croze MD, M Laé MD), and Department of Medical Oncology (A-S Hamy MD), Institut Curie, Paris, France; Department of Pathology, Université de Rouen Normandie, Rouen, France (M Laé); Department of Medical Oncology (Prof G S Sonke MD, T G Steenbruggen MD) and Department of Pathology (M van Seijen MD, Prof J Wesseling MD), Netherlands Cancer Institute, Amsterdam, Netherlands; Department of Medical Oncology, Instituto de Investigación Sanitaria Gregorio Marañón, Madrid, Spain (Prof M Martín MD, M del Monte-Millán BSc, S López-Tarruella MD); Department of Surgery (Prof J C Boughey MD), Department of Oncology (Prof M P Goetz MD), and Department of Health Sciences Research (T Hoskin MS), Mayo Clinic, Rochester, MN, USA; Department of Pathology and Translational Molecular Pathology (R Gould BSc, Prof W F Symmans MBChB) and Department of Breast Medical Oncology (Prof V Valero MD), The University of Texas MD Anderson Cancer Center, Houston, TX, USA; Department of Surgical Oncology, Roswell Park Cancer Institute, Buffalo, NY, USA (Prof S B Edge MD); Department of Oncology (J E Abraham PhD, Prof C Caldas MD, Prof H Earl MBBS, S-J Sammut MD) and Department of Histopathology (E Provenzano MBBS), University of Cambridge, Cambridge, UK; Diagnostic Development Program, Ontario Institute for Cancer Research, Toronto, Canada (Prof J M S Bartlett PhD); Deanery of Molecular, Genetic and Population Health Sciences, Edinburgh Cancer Research Centre, Edinburgh, UK (Prof J M S Bartlett); Department of Laboratory Medicine and Pathobiology, University of Toronto, Toronto, Ontario, Canada (Prof J M S Bartlett); Warwick Clinical Trials Unit, University of Warwick, Coventry, UK (Prof J Dunn PhD, L Hiller PhD); Department of Oncology (Prof L Hayward MRCP, Prof D Cameron MD, P Hall PhD) and Department of Pathology (J S Thomas MBBS, A Graham MBChB, L Mackintosh MBChB), Western General Hospital, Edinburgh, UK; Department of Pathology and Laboratory Medicine (Prof F Fan MD, Prof A K Godwin PhD) and Department of Medical Oncology (K Schwensen BS, Prof P Sharma MD), University of Kansas Medical Center, Kansas City, KS, USA; Department of Medicine, University of Pennsylvania, Philadelphia, PA, USA (Prof A M DeMichele MD); Department of Pathology (K Cole MD) and Department of Medical Oncology (Prof L Pusztai MD), Yale University, New Haven, CT, USA

## Abstract

**Background:**

Previous studies have independently validated the prognostic relevance of residual cancer burden (RCB) after neoadjuvant chemotherapy. We used results from several independent cohorts in a pooled patient-level analysis to evaluate the relationship of RCB with long-term prognosis across different phenotypic subtypes of breast cancer, to assess generalisability in a broad range of practice settings.

**Methods:**

In this pooled analysis, 12 institutes and trials in Europe and the USA were identified by personal communications with site investigators. We obtained participant-level RCB results, and data on clinical and pathological stage, tumour subtype and grade, and treatment and follow-up in November, 2019, from patients (aged ≥18 years) with primary stage I–III breast cancer treated with neoadjuvant chemotherapy followed by surgery. We assessed the association between the continuous RCB score and the primary study outcome, event-free survival, using mixed-effects Cox models with the incorporation of random RCB and cohort effects to account for between-study heterogeneity, and stratification to account for differences in baseline hazard across cancer subtypes defined by hormone receptor status and HER2 status. The association was further evaluated within each breast cancer subtype in multivariable analyses incorporating random RCB and cohort effects and adjustments for age and pretreatment clinical T category, nodal status, and tumour grade. Kaplan-Meier estimates of event-free survival at 3, 5, and 10 years were computed for each RCB class within each subtype.

**Findings:**

We analysed participant-level data from 5161 patients treated with neoadjuvant chemotherapy between Sept 12, 1994, and Feb 11, 2019. Median age was 49 years (IQR 20–80). 1164 event-free survival events occurred during follow-up (median follow-up 56 months [IQR 0–186]). RCB score was prognostic within each breast cancer subtype, with higher RCB score significantly associated with worse event-free survival. The univariable hazard ratio (HR) associated with one unit increase in RCB ranged from 1·55 (95% CI 1·41–1·71) for hormone receptor-positive, HER2-negative patients to 2·16 (1·79–2·61) for the hormone receptor-negative, HER2-positive group (with or without HER2-targeted therapy; p<0·0001 for all subtypes). RCB score remained prognostic for event-free survival in multivariable models adjusted for age, grade, T category, and nodal status at baseline: the adjusted HR ranged from 1·52 (1·36–1·69) in the hormone receptor-positive, HER2-negative group to 2·09 (1·73–2·53) in the hormone receptor-negative, HER2-positive group (p<0·0001 for all subtypes).

**Interpretation:**

RCB score and class were independently prognostic in all subtypes of breast cancer, and generalisable to multiple practice settings. Although variability in hormone receptor subtype definitions and treatment across patients are likely to affect prognostic performance, the association we observed between RCB and a patient’s residual risk suggests that prospective evaluation of RCB could be considered to become part of standard pathology reporting after neoadjuvant therapy.

## Introduction

Neoadjuvant chemotherapy was introduced for patients with locally advanced inoperable breast cancer in the late 1970s.^1^ Neoadjuvant chemotherapy is at least as effective as adjuvant therapy and has several additional advantages.^2^ Compared with adjuvant therapy, neoadjuvant therapy permits less extensive breast and axillary surgery by downstaging the tumour and allows monitoring of the treatment response, which provides important prognostic information. Pathological complete response (pCR) to neoadjuvant chemotherapy, defined as the absence of residual invasive disease in the breast and axilla, is strongly associated with improved long-term survival outcomes.^3–5^ The influential meta-analysis of the Collaborative Trials in Neoadjuvant Breast Cancer (CTNeoBC) showed that patients with pCR have improved event-free survival and overall survival, with the greatest prognostic value in patients with highly proliferative tumours.^4^ Consequently, the US Food and Drug Administration and European Medicines Agency issued initial guidance in 2012–14 for the use of pCR as a regulatory endpoint for accelerated approval of new drugs for neoadjuvant chemotherapy of breast cancer. Since these guidances were issued, contemporary trials have incorporated standardised pathological assessments of surgical resection specimens and validated pCR as a reliable prognostic marker.^6^ Increasingly, the presence or absence of residual disease is being used to guide adjuvant decisions following neoadjuvant chemotherapy.^7,8^

The binary outcome of pCR versus residual disease confers little information, offering no distinction among patients with varied amounts of residual disease. Furthermore, methods to evaluate surgical specimens and report residual disease have not been adequately standardised within pathology practice. The residual cancer burden (RCB) method, first described in 2007, was designed to address these shortcomings by providing a standard set of methods to evaluate and quantify the extent of residual disease in breast and axillary lymph nodes following neoadjuvant chemotherapy.^9^ It yields a continuous score, with pCR being the equivalent of an RCB score of zero. Empirically derived cutpoints are applied to the continuous score to define four RCB classes, from RCB-0 to RCB-3, that represent an increasing residual disease burden. RCB assessments are highly reproducible between pathologists;^10,11^ and both RCB and its classes have been validated as prognostic in single-institution studies^12–15^ and multicentre trials.^16–19^ However, individually, these cohorts are too small to obtain accurate estimates of prognosis related to RCB within the various subtypes of breast cancer. Therefore, we performed a pooled participant-level analysis of multiple clinical trials and cohorts to evaluate the overall association between RCB and long-term outcomes, with emphasis on the breast cancer subtypes defined by hormone receptor and HER2 receptor status. Our aim was to understand the prognostic value of RCB relative to pCR in the context of breast cancer subtypes, to optimise the interpretation of RCB and better inform patient management across a broad array of practice settings.

## Methods

### Study design and patient cohorts

In this pooled analysis, 12 institutes and trials in Europe and the USA were identified by personal communications with site investigators. For inclusion in this pooled analysis of participant-level data, trials or cohorts were required to include adult patients (aged ≥18 years) with primary stage I–III breast cancer (no positive sentinel lymph node biopsy, any phenotypic subtype) treated with neoadjuvant chemotherapy followed by surgery; and have available data for RCB, and follow-up data to evaluate the primary endpoint of event-free survival and the secondary endpoint of distant relapse-free survival. Data on Eastern Cooperative Oncology Group performance status was not available in all patients and thus was not collected. Investigators from institutions or trials who were known to have assessed and reported RCB in a pre-defined cohort were invited to participate between October, 2018, and April, 2019 (and all accepted); pooled data was finalised in November, 2019. Participating investigators representing 12 groups (four trials and eight clinical cohorts; appendix pp 2–3) from Europe and the USA provided individual patient data. We provide references or registration numbers for trials and cohorts when available.

The following trials were included: the I-SPY 1 trial,^17^ the I-SPY 2 trial,^18,20^ the ARTemis trial,^16^ and a trial led by the Instituto de Investigación Sanitaria Gregorio Marañón (IISGM; Madrid, Spain).^19^ Two of the trials included investigational therapies: the ARTemis study, in which bevacizumab was the investigational drug; and I-SPY 2, in which nine investigational drugs were adaptively randomised (4:1) against a concurrent control.^18,20^ I-SPY 1 and the IISGM trials were both observational, evaluating standard chemotherapies without any experimental arms.

The eight clinical cohorts were the MDACC cohort (MDACC-LAB98–240 and MDACC-LAB02–010 protocols) of the MD Anderson Cancer Center (Houston, TX, USA),^12^ the NEOREP cohort (CNIL declaration number 157270) from the Curie Institute (Paris, France),^15^ the triple-negative breast cancer PROGECT registry of the University of Kansas Medical Center (KUMC; Kansas City, KS, USA),^13^ the TransNEO cohort from the University of Cambridge (Cambridge, UK; European Genome-Phenome number EGAS00001004582), and cohorts from the Edinburgh Breast Unit at Western General Hospital (Edinburgh, UK; Edinburgh Cancer Information Programme Board reference number CIR21166), the Mayo Clinic (Rochester, MN, USA), the Netherlands Cancer Institute (Amsterdam, Netherlands),^14^ and Yale University (New Haven, CT, USA).

After neoadjuvant treatment and surgery, patients in each trial or cohort received adjuvant endocrine therapy, HER2 therapy, and locoregional radiotherapy, per their institution’s standard of care. For the remainder of this Article, we refer to all these trials and clinical cohorts as cohorts. Details on the cohorts, including eligibility criteria, type of consent, ethical approval, enrolment period, and patient characteristics, are provided in the appendix (pp 2–3). All patient identifiers were removed from data before the data were transferred and collated into a single dataset for the present analysis.

### Procedures

RCB was assessed by breast cancer pathologists at the treating centres (including DdC, ML, JeW, EP, JST, LM, AG, FF, KC, and WFS) trained in using the standard methods to evaluate and calculate RCB score and class.^9^ RCB was evaluated prospectively for five of the 12 cohorts (KUMC, I-SPY 2, IISGM, Mayo Clinic, and Yale cohorts), whereas RCB was determined in a retrospective review by the original investigators for the other seven cohorts (appendix pp 2–3). RCB values used in this analysis were based on reporting at the treating centre and were not centrally reviewed.

RCB (or RCB score) is calculated as a continuous variable. To aid in interpretation, cutpoints are applied to define four RCB classes indicating progressively larger residual disease burden: RCB-0 (RCB score 0, equivalent to pCR), RCB-1 (RCB score ≥0–1·36), RCB-2 (RCB score 1·37–3·28), and RCB-3 (RCB score >3·28).^9^

Evaluation of pretreatment histological grade was done at the treating institutions according to the Elston–Ellis modification of the Scarff–Bloom–Richardson grading system.^21^ Oestrogen receptor (encoded by *ESR1*) and progesterone receptor (encoded by *PGR*) status used in this analysis were as defined and provided by the institutions. Two cohorts (ARTemis and TransNEO) only recorded oestrogen receptor status and not progesterone receptor status. Thus, for our analysis, hormone receptor status was determined based on oestrogen and progesterone receptor status if both were available; or oestrogen receptor status alone if progesterone receptor status was not available. In the ARTemis trial, the TransNEO cohort, and Edinburgh cohort, hormone receptor status was defined as positive if the Allred score was 3 or higher. In other cohorts, hormone receptor status was defined by the percentage of cells that stained positive on immunohistochemistry at either a 1% or 10% threshold, depending on the institution. HER2 (*ERBB2*) status was determined according to international guidelines at all institutions.^22^ Hormone receptor and HER2 status were used to define four phenotypic subtypes (hormone receptor-negative, HER2-negative; hormone receptor-negative, HER2-positive; hormone receptor-positive, HER2-positive; and hormone receptor-positive, HER2-negative) for analysis. Treatment information, such as neoadjuvant HER2-targeted therapy use for HER2-positive patients, and histological-type data were also collected from the cohorts. Data collected in each cohort for the purposes of this study are summarised in the appendix (pp 2–3).

### Outcomes

The primary endpoint was event-free survival, adapted from the standardised definitions proposed in the CTNeoBC study, and measured as time from start of neoadjuvant treatment to the occurrence of an event.^4^ Any locoregional recurrence, distant recurrence, or death from any cause was considered as an event-free survival event, and patients without an event were censored at the date of last follow-up. The secondary endpoint was distant relapse-free survival, defined as time from start of neoadjuvant therapy to distant recurrence or death from any cause. Patients without an event were censored at the date of last follow-up. Follow-up was calculated from the start date of neoadjuvant chemotherapy.

### Statistical analysis

Baseline characteristics are presented as number and proportion for categorical variables and median (IQR) for continuous variables. IQR bounds were calculated with the formula: Q1–(1·5×IQR) and Q3+(1·5×IQR). Both the primary and secondary outcomes were assessed in all patients in the pooled analysis cohort. The association between RCB score and event-free survival and distant relapse-free survival in the pooled population was assessed with mixed-effects Cox models, which included random cohort and RCB effects to account for between-cohort heterogeneity, and stratification to account for differences in baseline hazard across biological breast cancer subtypes. The significance of the association was determined by the significance of the mean hazard ratio (HR) associated with a 1-unit increase in RCB score on a log-transformed scale, with a p value lower than 0·05 as the significance threshold. Similar mixed-effects models were used in prespecified subgroup analyses to assess the associations between RCB score and event-free survival within each breast cancer subtype. In addition, multivariable mixed-effects Cox analysis incorporating random cohort and RCB effects and adjusting for age, pretreatment T category (T0–1, T3, or T4 *vs* T2), pretreatment nodal status (positive *vs* negative), and pretreatment tumour grade (3 *vs* 1–2; all as fixed effects) as covariates were done (overall and within each subtype) to evaluate whether or not RCB remains significantly prognostic independent of these clinical covariates. We also evaluated associations within each participating cohort using fixed-effects univariable Cox models stratified by subtype. For the two HER2-positive subtypes, post-hoc analyses of patient subsets who received neoadjuvant HER2-targeted therapies in addition to neoadjuvant chemotherapy, which is now standard of care, was also performed. Results of the HER2-targeted subset are preferentially presented over results for the entire set of HER2-positive subtypes due to clinical relevance. In addition, to evaluate the non-linear effect of RCB on survival, we used B-splines with two degrees of freedom in our mixed-effects models and constructed relative event rate plots (with an RCB score of 0 as the reference) as a function of increasing RCB. Mixed-effects analysis was conducted with the coxme package in R (version 3.4.3). Kaplan-Meier plots of event-free survival and distant relapse-free survival by RCB class, overall and within breast cancer subtypes, were constructed with survival times truncated at 12 years (a time at which around 10% of the smallest patient group [RCB-1] remained at risk for an event); survival estimates at 3, 5, and 10 years were computed.

### Role of the funding source

The funder of the study had no role in study design, data collection, data analysis, data interpretation, or writing of the report.

## Results

**Figure 1:**
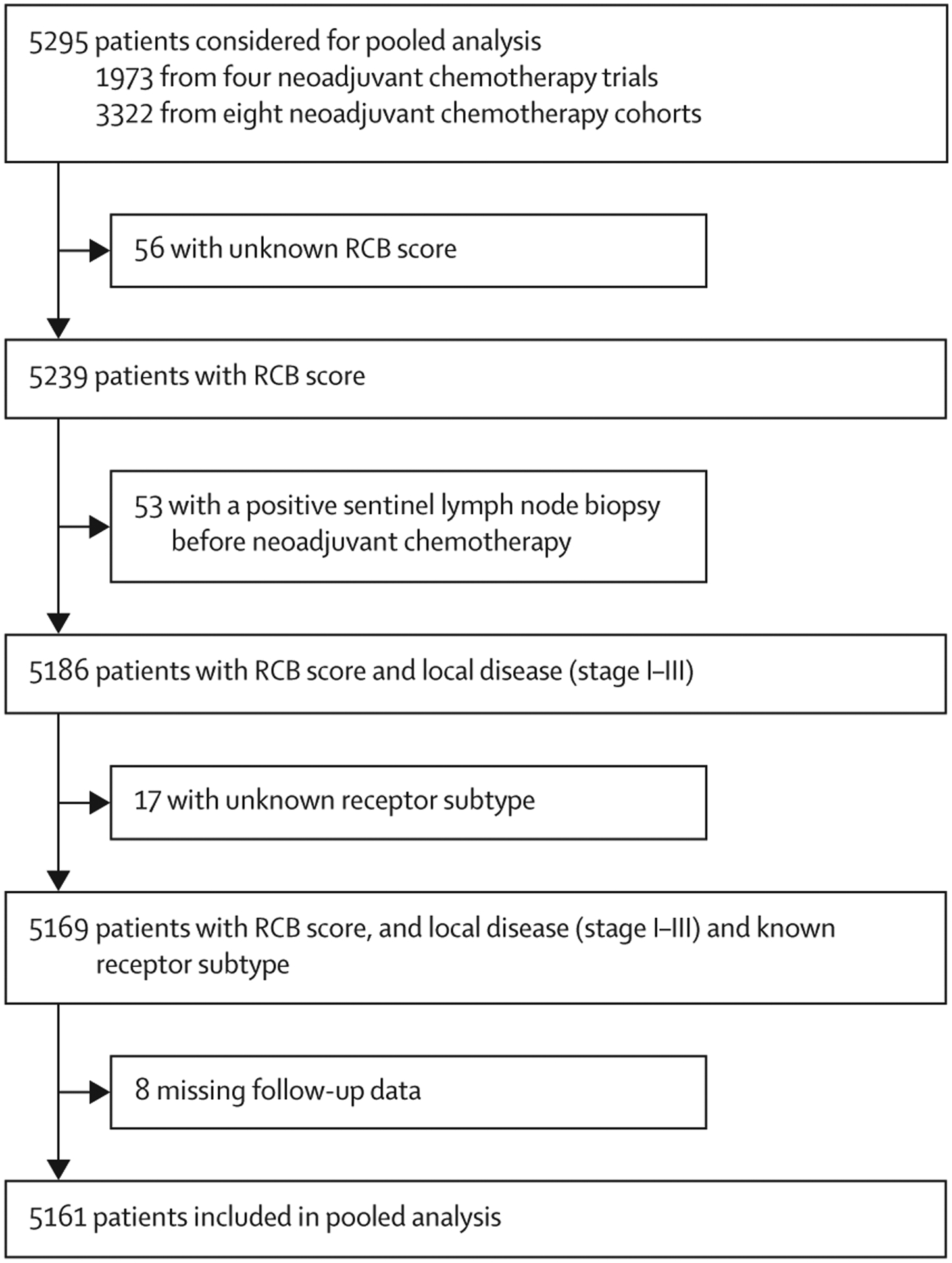
Study profile RCB=residual cancer burden.

5295 patients from 12 participating groups treated with neoadjuvant chemotherapy between Sept 12, 1994, and Feb 11, 2019 were identified for the pooled analysis. Patients with missing RCB score (n=56), a positive sentinel lymph node biopsy before neoadjuvant chemotherapy (n=53), unknown receptor subtype (n=17), or missing follow-up information (n=8) were excluded, yielding a total of 5161 eligible patients for analysis (figure 1).

Baseline patient and tumour characteristics, RCB class distribution, and follow-up information are summarised in table 1 for the overall population and by breast cancer subtype. In the overall population, median age was 49 years (IQR 20–80). 1774 (34·4%) of 5161 patients had hormone receptor-negative, HER2-negative tumours, 1430 (27·7%) had HER2-positive tumours (of whom 858 [60·0%] were hormone receptor-positive and 572 [40·0%] hormone receptor-negative) and 1957 (37·9%) had hormone receptor-positive, HER2-negative tumours. 1244 (87·0%) of the 1430 HER2-positive patients received neoadjuvant HER2-targeted therapy in addition to neoadjuvant chemotherapy. Overall, the proportions of patients in each RCB class were: 1676 (32·5%) of 5161 in RCB-0 (pCR), 662 (12·8%) in RCB-1, 2017 (39·1%) in RCB-2, and 806 (15·6%) in RCB-3 (table 1). Median follow-up was 56 months (IQR 0–186), with 1164 event-free survival events and 1072 distant relapse-free survival events.

**Figure 2:**
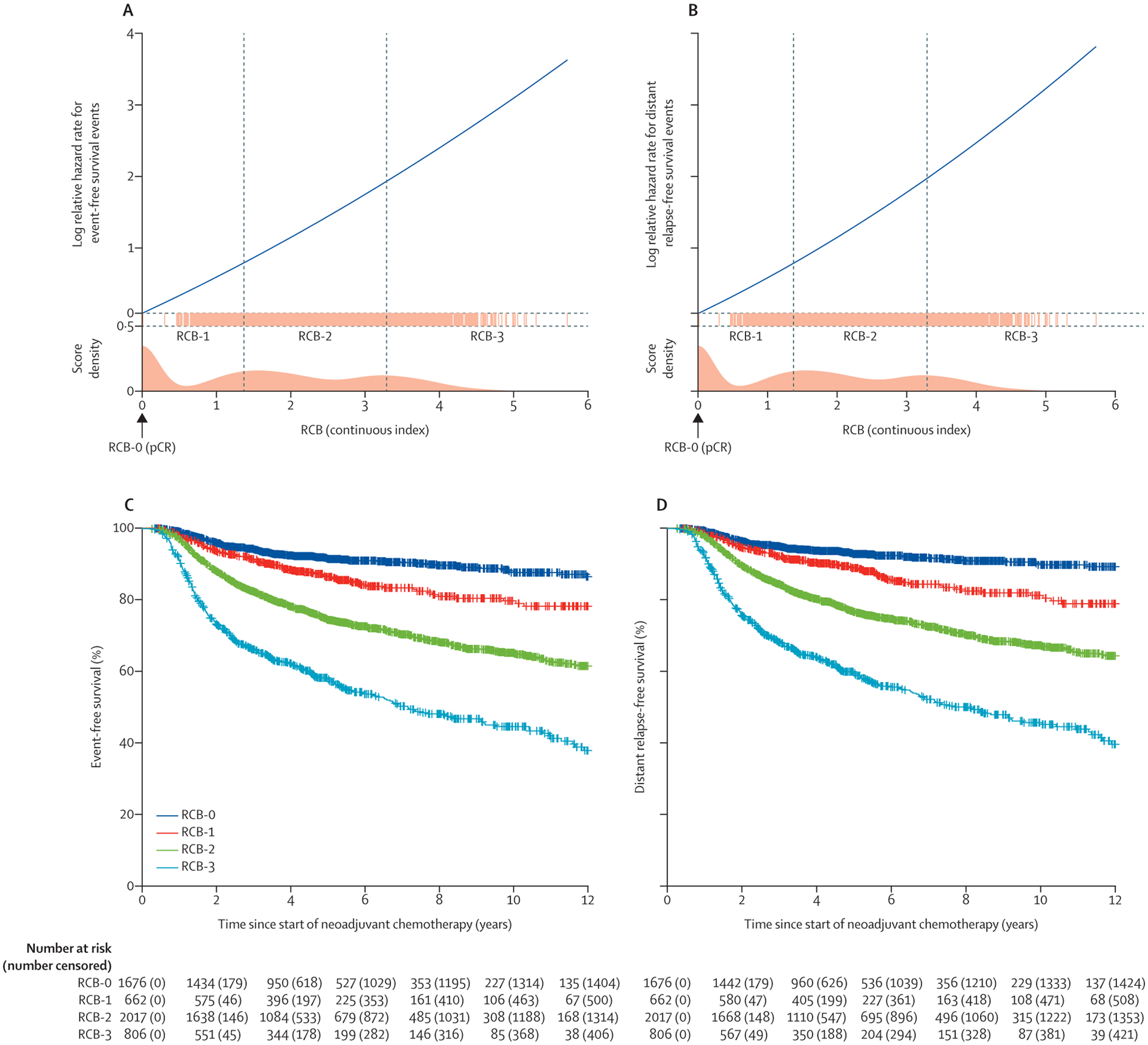
Prognostic value of RCB score and RCB class in the overall pooled analysis cohort Plots of log relative hazard rate for event-free survival events (A) and distant relapse-free survival events (B) as a function of RCB score. Splines approximation of RCB with two degrees of freedom was used to allow for non-linear effect. A log linear increase in relative hazard rate implies that the hazard ratio associated with change in RCB remains constant over the range of RCB. Thresholds for corresponding RCB classes (RCB-0 to RCB-3) are shown for reference (vertical dashed lines). Vertical bars represent all RCB scores recorded on a continuous scale. Kaplan-Meier plots of event-free survival (C) and distant relapse-free survival (D) stratified by RCB class. Crosses denote patients censored. RCB=residual cancer burden. pCR=pathological complete response.

In the overall population, increased RCB score was significantly associated with worse event-free survival and distant relapse-free survival overall in univariable analysis, with a HR per unit increase in RCB score of 1·82 (95% CI 1·73–1·91, p<0·0001) for event-free survival and 1·86 (1·76–1·97, p<0·0001) for distant relapse-free survival (appendix p 4). The log relative hazard rate (compared to RCB-0) for event-free survival and distant relapse-free survival events became larger with increasing RCB score, with a near-linear relationship for the pooled population (figure 2A, 2B). Similar associations with event-free survival and distant relapse-free survival were observed within each participating cohort (appendix p 9).

In multivariable analysis, associations between RCB and both event-free survival and distant relapse-free survival in the overall population remained significant when we adjusted for age, clinical tumour and nodal stage categories, and histological grade at baseline (event-free survival HR 1·69 [1·55–1·85], p<0·0001; distant relapse-free survival HR 1·75 [1·60–1·90], p<0·0001). Additionally, clinical T3 and T4 category and histological grade 3 were associated with significantly increased risk of event-free survival and distant relapse-free survival events, and node positivity was significantly associated with event-free survival events, in the multivariable model (table 2, appendix pp 7–8). RCB class was prognostic for both event-free survival (figure 2C) and distant relapse-free survival (figure 2D) in the overall population, with clear prognostic separation between each class.

Event-free survival estimates for patients within the RCB-0 class were 94% (95% CI 93–95) at 3 years, 91% (90–93) at 5 years, and 88% (85–90) at 10 years; compared with 91% (89–93), 86% (84–89), and 80% (76–84) for RCB-1; 82% (81–84), 74% (72–76), and 65% (62–68) for RCB-2; and 66% (63–70), 58% (54–62), and 45% (40–49) for RCB-3 (figure 2C, appendix pp 4–6). Similarly, distant relapse-free survival estimates were 95% (95% CI 94–96), 93% (91–94), and 90% (88–92) for RCB-0 at 3, 5, and 10 years; compared with 92% (90–94), 89% (86–91), and 81% (77–85) for RCB-1; 84% (83–86), 77% (75–79), and 67% (65–70) for RCB-2; and 68% (65–71), 60% (56–63), and 46% (41–51) for RCB-3 (figure 2D, appendix pp 4–6).

**Figure 3:**
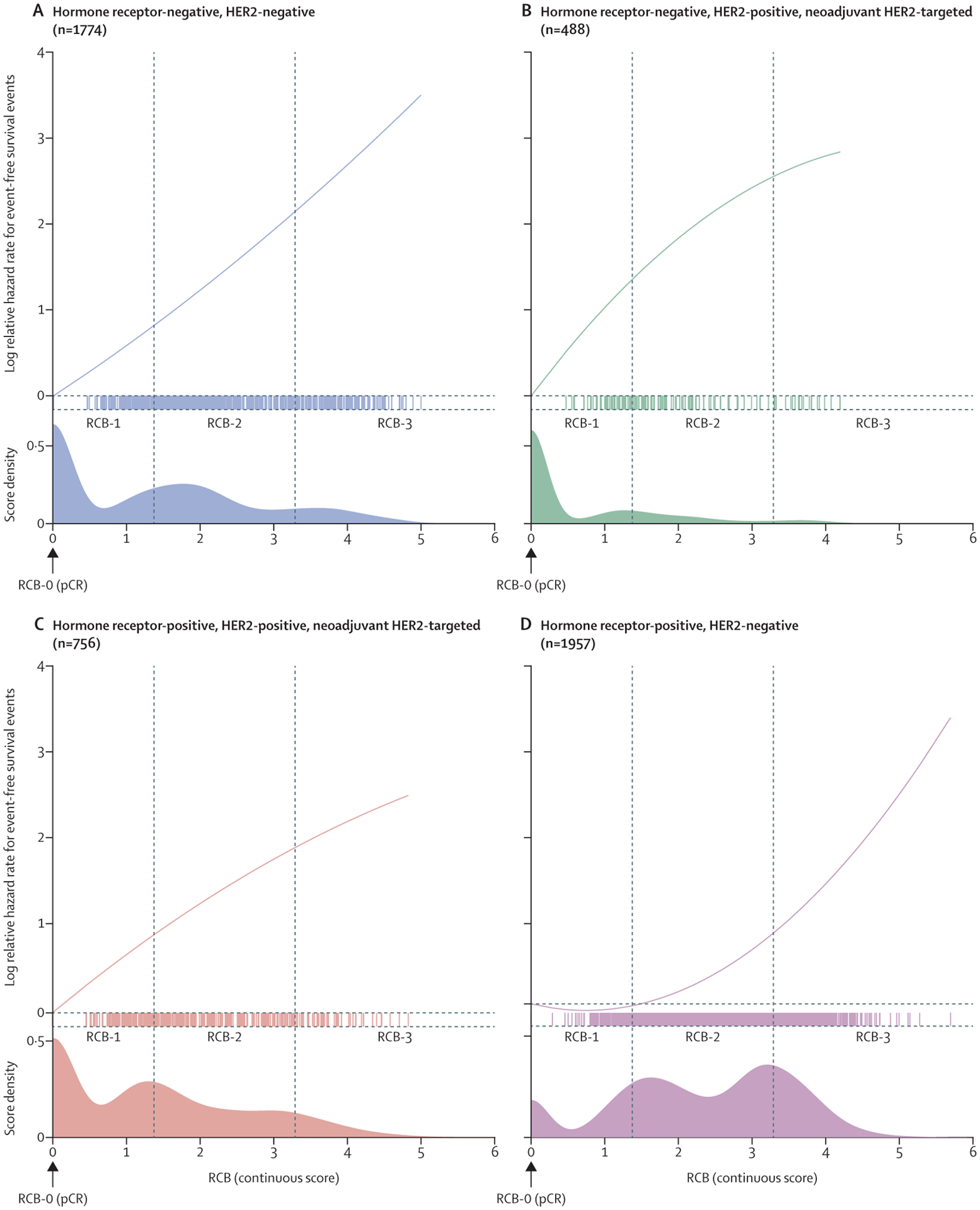
Prognostic value of RCB score within hormone receptor and HER2 subtypes Plots of log relative hazard rate for event-free survival events as a function of RCB score among breast cancer subtypes. For the two HER2-positive subtypes, plots of the subset of patients who received neoadjuvant HER2-targeted therapy are shown (plots for all HER2-positive patients, with or without HER2-targeted therapy, are presented in the appendix p 11). Splines approximation of RCB with two degrees of freedom was used to allow non-linear effect. A log linear increase in relative hazard rate implies that the hazard ratio associated with change in RCB remains constant over the range of RCB. Thresholds for corresponding RCB classes (RCB-0 to RCB-3) are shown for reference (vertical dashed lines). Vertical bars represent all RCB scores recorded on a continuous scale. RCB=residual cancer burden. pCR=pathological complete response.

Increased RCB score was significantly associated with worse event-free survival within all four breast cancer subtypes, with the HR associated with one unit increase in RCB score ranging from 1·55 (1·41–1·71) in the hormone receptor-positive, HER2-negative group to 2·16 (1·79–2·61) in the hormone receptor-negative, HER2-positive group (p<0·0001 for all subtypes; appendix pp 4–6). Similar findings were observed when considering only patients with hormone receptor-negative, HER2-positive tumours (488 of 572) or hormone receptor-positive, HER2-positive tumours (756 of 858) who received neoadjuvant HER2-targeted therapies with neoadjuvant chemotherapy (appendix pp 5–6). Increasing RCB was associated with a near-linear increase in log relative hazard rate among all breast cancer subtypes, except for the hormone receptor-positive, HER2-negative subtype, in which the log relative hazard rate remained near zero until an RCB score of around 1·5, close to the class threshold between RCB-1 and RCB-2 (figure 3; appendix p 11). The results were similar for distant relapse-free survival (appendix pp 4–6, 10–11).

In the multivariable analysis, RCB score remained a significant independent predictor of event-free survival and distant relapse-free survival in all breast cancer subtypes when we adjusted for baseline characteristics (table 2, appendix pp 7–8). For event-free survival, the adjusted HR associated with a one-unit increase in RCB score ranged from 1·52 (95% CI 1·36–1·69) in the hormone receptor-positive, HER2-negative group to 2·09 (1·73–2·53) in the hormone receptor-negative, HER2-positive group (p<0**·**0001 for all subtypes; appendix p 7). Similar results were observed for the distant relapse-free survival endpoint (appendix p 8).

**Figure 4:**
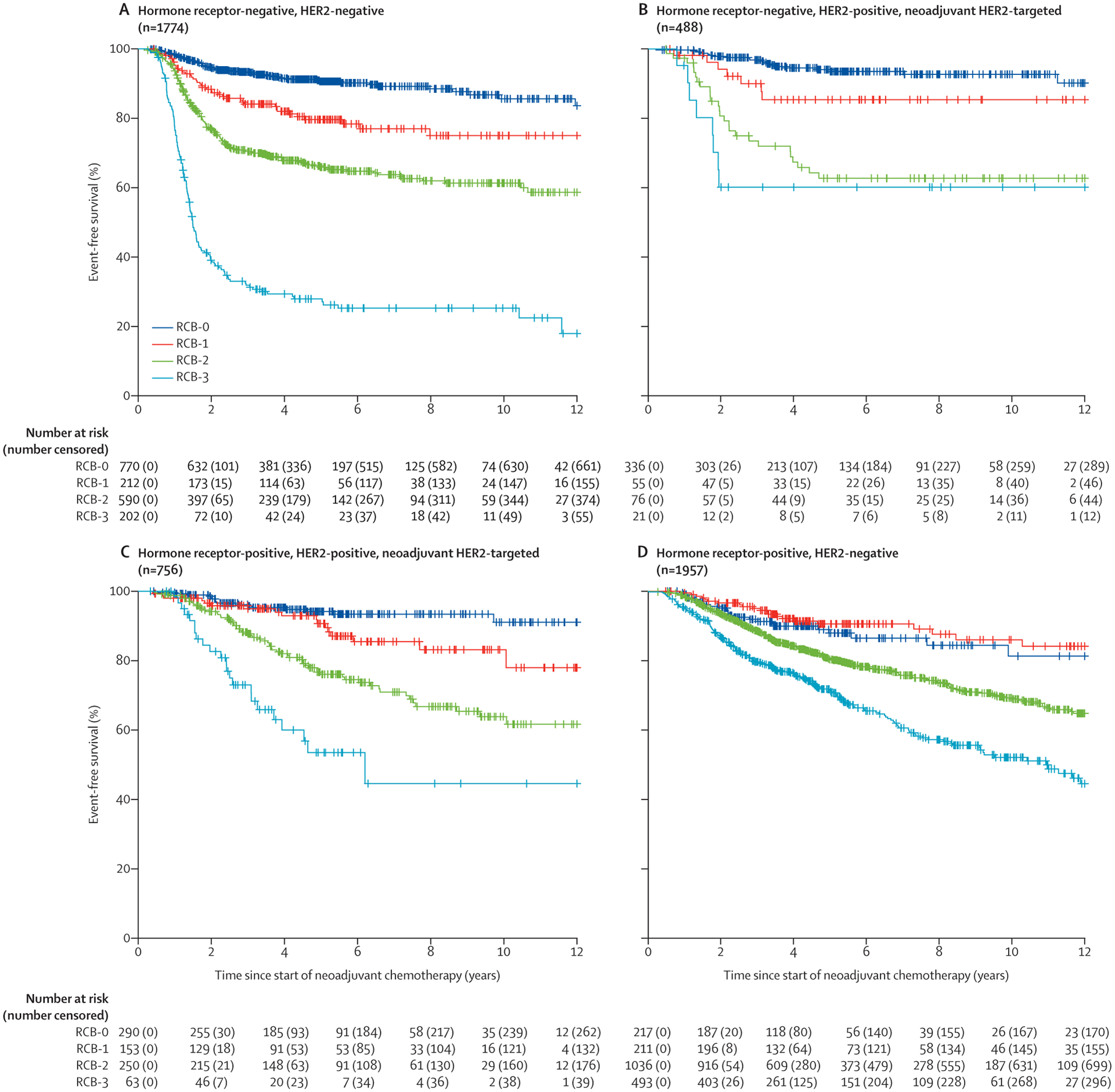
Prognostic value of RCB class for hormone receptor and HER2 subtypes Kaplan-Meier plots of event-free survival by RCB classes among breast cancer subtypes. For the two HER2-positive subtypes, plots of the subset of patients who received neoadjuvant HER2-targeted therapy are shown (plots for all HER2-positive patients, with or without HER2-targeted therapy, are presented in the appendix p 13). Crosses denote patients censored. RCB=residual cancer burden.

Despite differences in the distribution of RCB class between different breast cancer subtypes, we observed clear prognostic separation for event-free survival between patients with RCB-2 or RCB-3 disease and those who achieved a pCR (RCB-0) in all subtypes (figure 4, appendix pp 4–6, 13). Within the hormone receptor-negative, HER2-negative and hormone receptor-positive, HER2-positive subtypes, significant differences were also observed between the RCB-1 and RCB-0 groups (appendix pp 4–6). Notably, in the hormone receptor-positive, HER2-positive group who received HER2-targeted therapy, patients within the RCB-0 and RCB-1 classes showed similar event-free survival in the first 5 years (5-year event-free survival 94% [95% CI 91–97] and 91% [85–96], respectively) before their prognosis diverged; at 10 years, the event-free survival of RCB-0 patients was 91% (95% CI 86–97), compared with 83% (75–92) for RCB-1 patients (post-hoc analysis; figure 4C). Within the hormone receptor-positive, HER2-negative subtype, consistent with the non-linear relationship between event-free survival and continuous RCB, RCB-0 and RCB-1 patients had similar event-free survival (HR 0·97 [0·57–1·65], p=0·90; figure 4D, appendix pp 4–6). The characteristics of event-free survival events among hormone receptor-positive, HER2-negative RCB-0 patients are shown in the appendix (p 14). Results for the distant relapse-free survival endpoint were similar to those for event-free survival (appendix pp 4–6, 12).

## Discussion

In this pooled analysis, we showed that RCB is prognostic across 12 independent cohorts of patients, irrespective of pretreatment clinicopathological features and regardless of hormone receptor and HER2 subtype. At present, no universally adopted standard methodological approach is available for the pathological evaluation of response to neoadjuvant chemotherapy in breast cancer.^23^ More than 10 years ago, the degree of residual invasive disease in breast cancer was not believed to be of crucial importance for patient management, in part because mastectomy was the gold standard for patients with locally advanced breast cancer. Use of neoadjuvant chemotherapy increased as improved systemic therapies emerged and research evidence showed that breast conservation following neoadjuvant chemotherapy led to similar outcomes to mastectomy.^24^ Several studies have since shown the strong prognostic relationship between the presence or extent of residual disease and the risk of locoregional and distant recurrences.^9,10,17^ In this analysis, the number of event-free survival and distant relapse-free survival events was similar (1164 *vs* 1072), indicating that distant recurrences are the predominant risk for patients selected for neoadjuvant chemotherapy. Our definitions of event-free survival and distant relapse-free survival endpoints are consistent with the CTNeoBC meta-analysis^4^ and the standardised definitions for efficacy endpoints system (commonly known as STEEP), which recommends the date of first therapy as the starting point for time-to-event calculations.

Important aspects to the RCB method are that it provides both a standardised approach for pathological evaluation of post-treatment resection specimens and an algorithm that quantifies the extent of residual disease. Studies have reported highly reproducible measurements of RCB from different pathologists^10,11^ and the prognostic value of RCB has been validated in several single-centre studies and multicentre trials.^12–19^ Indeed, in this pooled analysis, we observed significant associations between RCB and event-free survival or distant metastasis-free survival in the overall population, within all breast cancer subtypes, and across all cohorts (except in the smallest cohort for event-free survival). Since our pooled cohorts represent a variety of clinical settings, this result implies a broad generalisability of the association between RCB and prognosis in the overall patient population and within each molecular subtype of breast cancer.

Importantly, the risk of a recurrence event increased with the extent of residual disease, regardless of breast cancer subtype. Use of RCB, therefore, adds prognostic information when pCR is not achieved. As more post-neoadjuvant (adjuvant) therapy options become available for patients with residual disease, a refined estimate of an individual’s risk of recurrence, based on their subtype and RCB, might be useful for informing decisions on adjuvant treatment selection. Interestingly, unlike in the hormone receptor-negative and hormone receptor-positive, HER2-positive subtypes, the increase in risk with RCB seems to be non-linear in the hormone receptor-positive, HER2-negative subtype. One potential reason for this relationship might be that the outcomes of some patients with hormone receptor-positive, HER2-negative cancer might not be dependent on response to neoadjuvant chemotherapy, but depend on the effects of the endocrine therapy that they usually receive for 5 years or longer.^25^ This result highlights the importance of subtypes in prognostication and suggests that use of RCB for recurrence risk prediction after neoadjuvant therapy should be performed within a subtype-specific context.

The weakest association between RCB and survival was in patients with hormone receptor-positive, HER2-negative tumours, among whom the RCB-0 and RCB-1 groups had similar event-free survival. This similarity in survival appeared to be driven by a few early recurrence events in the RCB-0 group (16 within the first 3 years). Five of these early recurrences occurred in the bevacizumab group of the ARTemis trial and might be attributable to a differential effect of bevacizumab, which increases pCR rates in the primary tumour but has less effect on micrometastatic disease.^26^ Variation in how hormone receptor positivity was defined across sites might also have an important role in the higher than expected early recurrence rates in the hormone receptor-positive, HER2-negative RCB-0 group. Three groups used Allred score, three groups defined positivity as more than 1% of cells with oestrogen receptor-positive staining, and others defined it as more than 10%, reflecting uncertainty on how to classify hormone receptor-low tumours. Five of the early recurrences in the hormone receptor-positive, HER2-negative RCB-0 group were observed in oestrogen receptor-negative (progesterone receptor-low) or oestrogen receptor-low (progesterone receptor-negative) cases. Whether these hormone receptor-low cases were more similar to hormone receptor-negative tumours or to their strongly hormone receptor-positive counterparts remains an unanswered question. Characterisation with various molecular subtypes, previously shown to associate with responsiveness to therapy and prognosis, might be informative.^27^

This study has several additional limitations. Patients received a range of neoadjuvant therapies (chemotherapy was given per each cohort institution’s standard of care with or without additional targeted therapies) and we did not control for treatment type or duration of treatment in this analysis. However, a previous analysis of the I-SPY 2 trial (cohort 2 in our analysis, appendix pp 2–3) suggests that the prognostic association of both pCR and RCB score is strong, regardless of type of chemotherapy-based treatment.^18,20^ Additionally, not all participating groups performed extensive metastatic workup as part of standard clinical care before neoadjuvant therapy, and the length of follow-up differed among the included cohorts. Furthermore, the proportion of lobular cancers in our study was less than the proportion of lobular cases in the overall breast cancer population,^28,29^ possibly reflecting the common belief among clinicians that lobular cancers do not respond well to neoadjuvant chemotherapy, and therefore clinicians do not select patients with lobular cancers for neoadjuvant therapy.

In this analysis, seven of 12 groups calculated RCB retrospectively, some reviewing specimens only when RCB or its components were unavailable in the original pathology report or only when residual disease was reported. pCR rate can decrease when the RCB method is incorporated into practice, possibly because a standardised and more focused pathological evaluation of the original tumour bed can identify residual disease that might otherwise have been missed.^30^ This is a shortcoming of retrospective pathology review because inaccurate sampling of the surgical specimen is the greatest potential source of residual disease misdiagnosed as pCR, and sampling accuracy cannot be determined by reviewing the slides. This limitation is particularly relevant in the hormone receptor-positive, HER2-negative subtype in which the proportion of diffuse disease is greater than in other subtypes,^31^ increasing the likelihood that sampling could affect the classification of RCB-0 and RCB-1. Additionally, only the most recent cohorts in our analysis used clips as standard practice to mark the sites, assuring that the original tumour bed was sampled. Prospective assessment of RCB, along with careful identification of the initial site of disease, might improve the overall prognostic performance of RCB. This approach should particularly apply in the setting of mastectomy, because it allows pathologists to identify the original site of disease using specimen radiographs and the clip placed during the biopsy at diagnosis for careful characterisation of the tumour bed.

Despite these limitations, the consistency of the prognostic importance of RCB across participating groups in our study highlights the generalisability of implementing and standardising the entire RCB methodology, from the stage of tissue acquisition to the final pathology assessment, across different countries, neoadjuvant chemotherapy treatments, and clinical settings. Altogether, our findings suggest that the RCB score has potential to be calibrated in a subtype-specific context to predict a patient’s residual risk after neoadjuvant chemotherapy in a prospective setting with standardised evaluation of post-treatment resection specimens. Given the increasing options for escalation and de-escalation of adjuvant therapy in the setting of residual disease, prospective evaluation of RCB as part of standard pathology reporting following neoadjuvant therapy might be warranted.

## Supplementary Material

1

## Figures and Tables

**Table 1:** Patient characteristics overall and by breast cancer subtype

	All participants (n=5161)	Hormone receptor-negative, HER2-negative (all patients; n=1774)	Hormone receptor-negative, HER2-positive (all patients; n=572)	Hormone receptor-negative, HER2-positive (neoadjuvant HER2-targeted; n=488)*	Hormone receptor-positive, HER2-positive (all patients; n=858)	Hormone receptor-positive, HER2-positive (neoadjuvant HER2-targeted; n=756)*	Hormone receptor-positive, HER2-negative (all patients; n=1957)
**Baseline characteristics**							
Age, years	49 (20–80)	49 (17–81)	51 (22–78)	51 (22–78)	48 (16–80)	48 (16–80)	49 (20–80)
T category							
0–1	466 (9·0%)	174 (9·8%)	56 (9·8%)	45 (9·2%)	84 (9·8%)	76 (10·1%)	152 (7·8%)
2	3139 (60·8%)	1132 (63·8%)	318 (55·6%)	277 (56·8%)	494 (57·6%)	444 (58·7%)	1195 (61·1%)
3	1026 (19·9%)	310 (17·5%)	138 (24·1%)	109 (22·3%)	172 (20·0%)	139 (18·4%)	406 (20·7%)
4	345 (6·7%)	106 (6·0%)	46 (8·0%)	43 (8·8%)	69 (8·0%)	59 (7·8%)	124 (6·3%)
Missing	185 (3·6%)	52 (2·9%)	14 (2·4%)	14 (2·9%)	39 (4·5%)	38 (5·0%)	80 (4·1%)
Node positivity	2780 (53·9%)	806 (45·4%)	360 (62·9%)	298 (61·1%)	499 (58·2%)	429 (56·7%)	1115 (57%)
Histological grade							
1	130 (2·5%)	16 (0·9%)	3 (0·5%)	3 (0·6%)	8 (0·9%)	6 (0·8%)	103 (5·3%)
2	1668 (32·7%)	270 (15·2%)	151 (26·4%)	130 (26·6%)	356 (41·5%)	313 (41·4%)	911 (46·6%)
3	2945 (57·1%)	1348 (76·0%)	378 (66·1%)	317 (65·0%)	437 (50·9%)	381 (50·4%)	782 (40%)
Missing	398 (8·1%)	140 (7·9%)	40 (7%)	38 (7·8%)	57 (6·6%)	56 (7·4%)	161 (8·2%)
Histological type							
Ductal or mixed ductal	4790 (92·8%)	1690 (95·3%)	542 (94·8%)	461 (94·5%)	814 (94·9%)	719 (95·1%)	1744 (89·1%)
Lobular	216 (4·2%)	19 (1·1%)	10 (1·7%)	9 (1·8%)	28 (3·3%)	24 (3·2%)	159 (8·1%)
Other	100 (1·9%)	42 (2·4%)	16 (2·8%)	14 (2·9%)	13 (1·5%)	10 (1·3%)	29 (1·5%)
Unknown or missing	55 (1·1%)	23 (1·3%)	4 (0·7%)	4 (0·8%)	3 (0·3%)	3 (0·4%)	25 (1·3%)
**Postneoadjuvant chemotherapy: RCB class**							
RCB-0	1676 (32·5%)	770 (43·4%)	376 (65·7%)	336 (68·9%)	313 (36·5%)	290 (38·4%)	217 (11·1%)
RCB-1	662 (12·8%)	212 (12·0%)	67 (11·7%)	55 (11·3%)	172 (20·1%)	153 (20·2%)	211 (10·8%)
RCB-2	2017 (39·1%)	590 (33·3%)	100 (17·5%)	76 (15·6%)	291 (33·9%)	250 (33·1%)	1036 (52·9%)
RCB-3	806 (15·6%)	202 (11·4%)	29 (5·1%)	21 (4·3%)	82 (9·6%)	63 (8·3%)	493 (25·2%)
**Follow-up information**							
Follow-up, months	56 (0–186)	45 (0–140)	69 (0–219)	65 (0–193)	64 (0–197)	61 (0–176)	58 (0–200)
Event-free survival events	1164	450	95	62	154	118	465
Distant relapse-free survival events	1072	417	79	53	135	100	441

Data are n, n (%), or median (IQR). Negative values for the lower IQR bound are truncated at 0. RCB=residual cancer burden.

* The subset who received neoadjuvant HER2-targeted therapy as neoadjuvant treatment in combination with chemotherapy.

**Table 2:** Multivariable mixed-effects Cox models of event-free survival as a function of RCB

	All patients (n=4607)*	Hormone receptor-negative, HER2-negative (all patients; n=1585)*	Hormone receptor-negative, HER2-positive (all patients; n=522)*	Hormone receptor-negative, HER2-positive (neoadjuvant HER2-targeted; n=440)*†	Hormone receptor-positive, HER2-positive (all patients; n=773)*	Hormone receptor-positive, HER2-positive (neoadjuvant HER2-targeted; n=674)*†	Hormone receptor-positive, HER2-negative (all patients; n=1727)*
RCB	1·69 (1·55–1·85)‡	1·93 (1·74–2·13)‡	2·09 (1·73–2·53)‡	2·10 (1·68–2·62)‡	1·66 (1·45–1·90)‡	1·69 (1·45–1·97)‡	1·52 (1·36–1·69)‡
Age	1·00 (0·99–1·00)	0·99 (0·98–1·00)‡	1·00 (0·98–1·02)	1·00 (0·97–1·03)	1·00 (0·99–1·02)	1·00 (0·98–1·02)	1·00 (0·99–1·01)
T category (reference: T2)§							
T0–1	1·08 (0·85–1·37)	1·05 (0·69–1·60)	1·99 (1·00–3·96)	2·46 (1·03–5·87)‡	0·80 (0·40–1·61)	0·50 (0·20–1·26)	1·01 (0·69–1·46)
T3	1·28 (1·10–1·49)‡	1·73 (1·37–2·18)‡	1·60 (0·95–2·69)	1·64 (0·83–3·24)	1·02 (0·66–1·56)	0·88 (0·53–1·48)	1·08 (0·85–1·37)
T4	1·89 (1·55–2·31)‡	1·43 (1·02–2·01)‡	1·27 (0·60–2·68)	2·39 (1·02–5·58)‡	3·23 (2·07–5·03)‡	2·98 (1·81–4·90)‡	2·11 (1·53–2·91)‡
Nodal status (reference: node negativity)							
Node positivity	1·15 (1·00–1·32)‡	1·17 (0·94–1·44)	0·87 (0·52–1·45)	0·72 (0·38–1·35)	1·25 (0·84–1·86)	1·34 (0·85–2·11)	1·30 (1·04–1·62)‡
Grade (reference: grade 1–2)							
Grade 3	1·51 (1·33–1·72)‡	1·09 (0·85–1·40)	0·96 (0·58–1·59)	0·86 (0·46–1·63)	0·76 (0·55–1·06)	0·68 (0·46–0·99)‡	1·55 (1·27–1·89)‡

RCB was analysed as a continuous score, adjusting for age and pretreatment T category, nodal status, and grade (as fixed effects). Hazard ratios (95% CIs) are shown. All p values are shown in the appendix (p 7). RCB=residual cancer burden.

* Patients with complete covariate data.

† The subset who received neoadjuvant HER2-targeted therapy as neoadjuvant treatment in combination with chemotherapy.

‡ Indicates significant p values less than 0·05.

§ T2 was used as the reference category due to the small sample size of the T0–1 group (particularly within the HER2-positive subtypes) in view of concern for the stability of the hazard ratio estimates.

## Data Availability

Data used in this study were made available under contract between the different institutes and groups and University of California, San Francisco (San Francisco, CA, USA). Agreements between the European and US institutions were based on the EU General Data Protection Regulation. Requests for datasets should be made to the original investigators from each cohort or trial within the pooled analysis (appendix p 15).

## Research in context

### Evidence before this study

The seminal meta-analysis of the Collaborative Trials in Neoadjuvant Breast Cancer working group published in 2014 showed that on an individual level, achieving a pathological complete response (pCR) following neoadjuvant chemotherapy was associated with improved long-term survival outcomes.On Sept 13, 2021, a search of the PubMed database using the term “pathological complete response and breast cancer prognosis” yielded 1531 published articles between Jan 1, 2014, and Dec 31, 2019. However, pCR does not provide distinction among patients with residual disease. The residual cancer burden (RCB) method was proposed in 2007 as a standardised methodological approach to evaluate and quantitate the extent of residual disease in breast and axillary lymph nodes following neoadjuvant chemotherapy. A further search of the PubMed database (Sept 13, 2021), using the term “residual cancer burden and breast cancer prognosis” identified 166 articles published between Jan 1, 2007, and Dec 31, 2019. Among these articles, single-institution studies and multicentre trials had validated RCB as a prognostic score in breast cancer.

### Added value of this study

Individually, the cohorts in previous studies evaluating the prognostic value of RCB are too small to obtain accurate estimates within the various molecular subtypes of breast cancer. By assembling a pooled cohort of 5161 patients across 12 participating groups from Europe and the USA representing a variety of clinical settings, our study was able to validate the prognostic value of RCB overall and within each hormone receptor and HER2 subtype, with regard to event-free survival and distant relapse-free survival. In addition, by evaluating RCB as a continuous measure in a model that allows for non-linear effect within each subtype, we were able to further characterise how risk of recurrence or death changes with increasing RCB and compare this relationship between different breast cancer subtypes.

### Implications of all the available evidence

The prognostic importance of pCR (equivalent to RCB score 0) is well established. RCB score adds substantially to the binary assessment of pCR versus residual disease in predicting long-term survival. The observed prognostic consistency of RCB collected across different countries and clinical settings highlights the generalisability of implementing the RCB methodology. RCB score has the potential to be used in a subtype-specific context to predict a patient’s residual risk after neoadjuvant chemotherapy in a prospective setting with standardised evaluation of post-treatment resection specimens, especially given the increasing options for adjuvant therapy in the setting of residual disease.
