# Supplementary material for: Residual cancer burden after neoadjuvant chemotherapy and long-term survival outcomes in breast cancer: a multicentre pooled analysis of 5161 patients
Source: Lancet Oncol. Author manuscript; Available in PMC 2022 Sep 8. (PMC9455620; doi:10.1016/S1470-2045(21)00589-1)
Supplement: 1 [file NIHMS1833556-supplement-1.pdf]

THE LANCET
Oncology

Supplementary appendix

This appendix formed part of the original submission and has been peer reviewed. We post it as supplied by the authors.

Supplement to: Yau C, Osdoit M, van der Noordaa M, et al. Residual cancer burden after neoadjuvant chemotherapy and long-term survival outcomes in breast cancer: a multicentre pooled analysis of 5161 patients. *Lancet Oncol* 2021; published online Dec 10. https://doi.org/10.1016/S1470-2045(21)00589-1.

Supplementary Appendix

Yau et al.

Residual Cancer Burden after neoadjuvant chemotherapy and long-term survival outcomes in breast cancer: a multi-center pooled analysis across 5161 patients

CONTENTS

Table S1. Cohort characteristics and patient characteristics by cohort ... 2

Table S2. Prognostic value of RCB and RCB class overall and within subtype in a univariate analysis showing hazard ratio associated with 1 unit increase in RCB score, Kaplan Meier estimates at 3, 5 and 10 years, and hazard ratio of RCB class relative to pCR for EFS and DRFS endpoints with 95% confidence intervals. ... 4

Table S3. Multivariate mixed effect Cox models of EFS as a function of RCB as a continuous index, adjusting for age, pre-treatment T category, pre-treatment N status and grade (as fixed effects) ... 7

Table S4. Multivariate mixed effect Cox models of DRFS as a function of RCB as a continuous index, adjusting for age, pre-treatment T category, pre-treatment N status and grade (as fixed effects) ... 8

Figure S1. Prognostic value of RCB overall and by site. ... 9

Figure S2. Prognostic value of RCB within HR/HER2 defined subtypes. ... 10

Figure S3. Prognostic value of RCB within HER2-positive subtypes ... 11

Figure S4. Prognostic value of RCB classes within HR/HER2 defined subtypes. ... 11

Figure S5. Prognostic value of RCB classes within HER2-positive subtypes ... 113

Table S5. Characteristics of EFS events among hormone-receptor positive HER2 negative RCB-0 patients. ... 14

Table S6. List of contacts for trials/cohorts within the pooled analysis. ... 15

Table S1. Cohort characteristics and patient characteristics by cohort

	Cohort 1	Cohort 2	Cohort 3	Cohort 4	Cohort 5	Cohort 6	Cohort 7	Cohort 8	Cohort 9	Cohort 10	Cohort 11	Cohort 12
Cohort Name	I-SPY 1	I-SPY 2	MDACC	Curie	IISGM	KUMC	TransNeo	Yale	Edinburgh	ARTemis	NKI	Mayo Clinic
N	196	916	1010	717	223	208	161	176	408	575	263	308
Type of study	Trial	Trial	Cohort	Cohort	Trial	Cohort	Cohort	Cohort	Cohort	Trial	Cohort	Cohort
Ethical Approval	IRB at each trial site (ACRIN 6657/CALGB 150012 and CALGB 150007)	IRB at each trial site (NCT01042379)	IRB protocols LAB98-240, LAB02-010	CNIL declaration number 157270	Ethnical Board at each participating institution (NCT 01560663)	IRB at each participating institution (NCT02302742)	REC reference 12/EE/0484	IRB Protocol ID 2000025353	Edinburgh Cancer Information Programme Board reference number CIR21166	Multi-Centre Research Ethics Committee and research ethics committees at each trial site	IRB number IRBd19089/ CFMPB611	IRB Protocol Number 15-003703
Experimental target therapy	No	Yes	No	No	No	No	No	No	No	Yes	No	No
Inclusion criteria	>3cm by clinical exam or imaging<br>M0	>2.5cm by clinical exam or imaging<br>M0<br>HR- or HER2+ or MammaPrint high risk	Stage I to III	cT1-3 N0-3 M0	HR-HER2-<br>cT1c-4 N1-3 M0	HR-HER2-<br>Stage I to III	cT0-4 N0-3 M0<br>Received NAC	Tx Nx M0	Tx Nx M0	HER2-<br>cT2-4 N0-3 M0	Stage II to III	Tx Nx M0
Exclusion criteria			Inflammatory BC	Inflammatory BC, Multicentric, Bilateral BC							Inflammatory BC	Bilateral BC
Type of consent	Written	Written	IRB approval provided waiver of individual consent	CNIL approval provided waiver of individual consent	Written	Written	Written	Approval to use deidentified information	Written	Written	Waiver because of deidentified data	IRB approval provided waiver of individual consent
Enrollment Period *	06/12/2002 - 03/01/2006	04/12/2010 - 10/27/2016	09/12/1994 - 10/19/2011	01/29/2002 - 05/02/2012	07/22/2010 - 02/11/2019	04/23/2008 - 03/23/2018	03/15/2013 - 03/04/2017	03/20/2005 - 06/19/2017	09/19/2012 - 08/22/2018	05/08/2009 - 01/15/2013	12/08/2004 - 03/15/2016	03/09/2009 - 08/05/2016
Pathologist Training	MDACC	MDACC	MDACC	Personal	MDACC Video	MDACC Video	Personal	MDACC Video	MDACC Video	Personal	MDACC Video	MDACC Video
Timing of RCB calculation	Retrospective	Prospective	Retrospective	Retrospective	Prospective	Prospective	Retrospective	Prospective	Retrospective	Retrospective	Retrospective	Prospective
**Baseline Characteristics**												
Age, Median (IQR)	49 (12)	49 (15)	50 (16)	48 (15)	52 (17)	51 (18)	51 (16)	48 (17)	50 (16)	47 (12)	48 (15)	51 (18)
Baseline T category, N (%)												
0/1	5 (2.55%)	30 (3.92%)	124 (12.27%)	42 (5.86%)	26 (11.66%)	61 (29.32%)	12 (7.45%)	46 (26.14%)	27 (6.62%)	28 (4.87%)	29 (11.03%)	36 (11.69%)
2	73 (37.24%)	511 (66.88%)	601 (59.5%)	489 (68.2%)	129 (57.85%)	122 (58.65%)	99 (61.49%)	104 (59.09%)	265 (64.95%)	406 (70.61%)	164 (62.36%)	176 (57.14%)
3	94 (47.96%)	189 (24.74%)	172 (17.03%)	186 (25.94%)	37 (16.59%)	18 (8.65%)	41 (25.47%)	22 (12.5%)	57 (13.97%)	75 (13.04%)	67 (25.48%)	68 (22.08%)
4	22 (11.22%)	34 (44.5%)	113 (11.19%)	0	31 (13.9%)	7 (3.37%)	8 (4.97%)	4 (2.27%)	58 (14.22%)	39 (6.78%)	2 (0.76%)	27 (8.77%)
Missing	2 (1.02%)	152 (16.94%)	0	0	0	0	1 (0.62%)	0	1 (0.25%)	27 (4.70%)	1 (0.38%)	1 (0.32%)
Baseline node positive, N (%)	123 (63.08%)	356 (48.04%)	643 (63.66%)	434 (60.61%)	107 (48.2%)	75 (36.76%)	85 (52.8%)	99 (56.9%)	204 (50.12%)	288 (50.09%)	189 (71.86%)	177 (57.47%)
Pre-treatment Histological Grade, N (%)												
I	15 (7.65%)	8 (0.87%)	57 (5.64%)	16 (2.23%)	4 (1.79%)	1 (0.48%)	0 (0%)	4 (2.27%)	3 (0.74%)	12 (2.09%)	3 (1.14%)	7 (2.27%)
II	90 (45.92%)	184 (20.09%)	403 (39.9%)	195 (27.2%)	56 (25.11%)	28 (13.46%)	63 (39.13%)	85 (48.3%)	168 (41.18%)	201 (34.96%)	113 (42.97%)	102 (33.12%)

III	88 (44.9%)	435 (47.49%)	550 (54.46%)	490 (68.34%)	151 (67.71%)	177 (85.1%)	98 (60.87%)	85 (48.3%)	237 (58.09%)	306 (53.22%)	131 (49.81%)	197 (63.96%)
Missing	3 (1.53%)	289 (31.55%)	0 (0%)	16 (2.23%)	12 (5.38%)	2 (0.96%)	0 (0%)	2 (1.14%)	0 (0%)	56 (9.74%)	16 (6.08%)	2 (0.65%)
Phenotypic HR/HER2 Subtype, N (%)												
HR-HER2-	47 (23.98%)	313 (34.17%)	213 (21.09%)	319 (44.49%)	223 (100%)	208 (100%)	37 (22.98%)	50 (28.41%)	105 (25.74%)	191 (33.22%)	0	68 (22.08%)
HR-HER2+	32 (16.33%)	87 (9.5%)	141 (13.96%)	72 (10.04%)	0	0	14 (8.7%)	23 (13.07%)	41 (10.05%)	0	123 (46.77%)	39 (12.66%)
HR+HER2+	30 (15.31%)	169 (18.45%)	160 (15.84%)	104 (14.5%)	0	0	47 (29.19%)	39 (22.16%)	91 (22.30%)	0	140 (53.23%)	78 (25.32%)
HR+HER2-	87 (44.39%)	347 (37.88%)	496 (49.11%)	222 (30.96%)	0	0	63 (39.13%)	64 (36.36%)	171 (41.91%)	384 (66.78%)	0	123 (39.94%)
HER2+ subset receiving neoadjuvant HER2-targeted therapy (together with NAC)												
HR-HER2+, N (%)	10 (31.2%)	87 (100%)	94 (66.7%)	59 (81.9%)	NA	NA	14 (100%)	21 (91.3%)	41 (100%)	NA	123 (100%)	39 (100%)
HR+HER2+, N (%)	7 (23.3%)	169 (100%)	104 (65%)	87 (83.7%)	NA	NA	47 (100%)	34 (87.2%)	91 (100%)	NA	140 (100%)	77 (98.7%)
Histologic Type												
Ductal or mixed ductal	161 (82.1%)	859 (93.8%)	956 (94.7%)	660 (92.1%)	223 (100%)	202 (97.1%)	143 (88.8%)	171 (97.2%)	385 (94.4%)	487 (84.7%)	244 (92.8%)	299 (97.1%)
Lobular	17 (8.7%)	30 (3.3%)	45 (4.5%)	21 (2.9%)	0 (0%)	1 (0.5%)	7 (4.3%)	3 (1.7%)	22 (5.4%)	51 (8.9%)	12 (4.6%)	7 (2.3%)
Other	16 (8.2%)	5 (0.5%)	9 (0.9%)	32 (4.5%)	0 (0%)	1 (0.5%)	11 (6.8%)	2 (1.1%)	0 (0%)	15 (2.6%)	7 (2.7%)	2 (0.6%)
Unknown or missing	2 (1%)	22 (2.4%)	0 (0%)	4 (0.6%)	0 (0%)	4 (1.9%)	0 (0%)	0 (0%)	1 (0.2%)	22 (3.8%)	0 (0%)	0 (0%)
**Post Neoadjuvant Chemotherapy: RCB Classes, N (%)**												
RCB-0	54 (27.55%)	333 (36.35%)	226 (22.38%)	202 (28.17%)	126 (56.5%)	126 (60.58%)	42 (26.09%)	46 (26.14%)	122 (29.9%)	119 (20.7%)	155 (58.94%)	125 (40.58%)
RCB-I	18 (9.18%)	124 (13.54%)	145 (14.36%)	65 (9.07%)	22 (9.87%)	20 (9.62%)	25 (15.53%)	31 (17.61%)	62(15.2%)	87 (15.13%)	37 (14.07%)	26 (8.44%)
RCB-II	85 (43.37%)	319 (34.83%)	455 (45.05%)	310 (43.24%)	61 (27.35%)	52 (25%)	66 (40.99%)	68 (38.64%)	159 (38.97%)	284 (49.39%)	59 (22.43%)	99 (32.14%)
RCB-III	39 (19.9%)	140 (15.28%)	184 (18.22%)	140 (19.53%)	14 (6.28%)	10 (4.81%)	28 (17.39%)	31 (17.61%)	65 (15.93%)	85 (14.78%)	12 (4.56%)	58 (18.83%)
**Follow-up Information**												
Median follow-up (IQR) (months)	85 (38)	46 (29)	125 (79)	99 (51)	36 (36)	32 (24)	43 (25)	67 (43)	32 (31)	41 (22)	71 (51)	35 (33)
EFS Events	67	177	316	205	41	24	21	30	81	131	31	40
DRFS Events	63	149	308	188	38	22	17	24	77	123	27	36

* Date of chemotherapy start of the first and last patient in the cohort were used as the approximation of the enrollment period

Table S2. Prognostic value of RCB and RCB class overall and within subtype in a univariate analysis showing hazard ratio associated with 1 unit increase in RCB score, Kaplan Meier estimates at 3, 5 and 10 years, and hazard ratio of RCB class relative to pCR for EFS and DRFS endpoints with 95% confidence intervals.

		EFS					DRFS				
		KM Estimates (95% CI)			Hazard Ratio (95% CI)	p	KM Estimates (95% CI)			Hazard Ratio (95% CI)	p
	N (%)	at 3yr	at 5yr	at 10yr			at 3yr	at 5yr	at 10yr		
**Overall (5161)**											
RCB (continuous index)					1.82 (1.73-1.91)	<0.00001				1.86 (1.76-1.97)	<0.00001
RCB Class											
RCB-0	1676 (32%)	94% (93%-95%)	91% (90%-93%)	88% (85%-90%)	Ref		95% (94%-96%)	93% (91%-94%)	90% (88%-92%)	Ref	
RCB-I	662 (13%)	91% (89%-93%)	86% (84%-89%)	80% (76%-84%)	1.99 (1.54-2.57)	<0.00001	92% (90%-94%)	89% (86%-91%)	81% (77%-85%)	2.09 (1.59-2.74)	<0.00001
RCB-II	2017 (39%)	82% (81%-84%)	74% (72%-76%)	65% (62%-68%)	4.01 (3.31-4.86)	<0.00001	84% (83%-86%)	77% (75%-79%)	67% (65%-70%)	4.14 (3.37-5.08)	<0.00001
RCB-III	806 (16%)	66% (63%-70%)	58% (54%-62%)	45% (40%-49%)	9.10 (7.41-11.18)	<0.00001	68% (65%-71%)	60% (56%-63%)	46% (41%-51%)	9.69 (7.79-12.06)	<0.00001
**HR-HER2- (1774)**											
RCB (continuous index)					1.98 (1.82-2.15)	<0.00001				2.02 (1.84-2.21)	<0.00001
RCB Class											
RCB-0	770 (43%)	93% (92%-95%)	91% (88%-93%)	86% (81%-90%)	Ref		94% (93%-96%)	92% (89%-94%)	87% (83%-91%)	Ref	
RCB-I	212 (12%)	84% (79%-89%)	80% (74%-86%)	75% (68%-83%)	2.16 (1.48-3.15)	0.00007	85% (80%-90%)	82% (76%-88%)	77% (70%-85%)	2.23 (1.5-3.32)	0.00007
RCB-II	590 (33%)	70% (67%-74%)	66% (62%-70%)	61% (57%-66%)	3.94 (3-5.18)	<0.00001	74% (70%-77%)	68% (64%-73%)	63% (58%-68%)	4.01 (3.01-5.35)	<0.00001
RCB-III	202 (11%)	32% (26%-39%)	28% (22%-35%)	25% (19%-33%)	13.04 (9.8-17.35)	<0.00001	34% (28%-42%)	29% (24%-37%)	27% (21%-34%)	13.63 (10.09-18.41)	<0.00001
**HR-HER2+ (572)**											
RCB (continuous index)					2.16 (1.79-2.61)	<0.00001				2.10 (1.76-2.5)	<0.00001
RCB Class											
RCB-0	376 (66%)	96% (94%-98%)	93% (90%-96%)	91% (87%-95%)	Ref		97% (95%-99%)	94% (92%-97%)	93% (90%-96%)	Ref	

RCB-I	67 (12%)	90% (83%-98%)	85% (76%-94%)	81% (71%-93%)	**2.05 (0.99-4.23)**	0.052	90% (83%-98%)	85% (76%-94%)	81% (71%-93%)	2.52 (1.2-5.31)	0.015
RCB-II	100 (17%)	71% (62%-80%)	58% (49%-69%)	55% (45%-67%)	7.08 (4.38-11.46)	<0.00001	78% (70%-87%)	68% (59%-78%)	66% (56%-76%)	6.23 (3.64-10.66)	<0.00001
RCB-III	29 (5%)	57% (42%-79%)	48% (33%-72%)	48% (33%-72%)	10.46 (5.44-20.13)	<0.00001	60% (45%-82%)	51% (35%-75%)	51% (35%-75%)	11.73 (5.87-23.43)	<0.00001
**HR-HER2+ (Received Neoadjuvant HER2-targed therapy) (488)**											
RCB (continuous index)					2.13 (1.71-2.66)	<0.00001				2.14 (1.71-2.67)	<0.00001
RCB Class											
RCB-0	336 (69%)	97% (95%-99%)	94% (91%-97%)	93% (89%-96%)	Ref		97% (95%-99%)	95% (92%-98%)	94% (91%-97%)	Ref	
RCB-I	55 (11%)	90% (82%-99%)	85% (76%-96%)	85% (76%-96%)	**2.26 (0.95-5.36)**	0.064	90% (82%-99%)	85% (76%-96%)	85% (76%-96%)	2.81 (1.15-6.84)	0.023
RCB-II	76 (16%)	73% (64%-84%)	63% (52%-75%)	63% (52%-75%)	7.04 (3.93-12.6)	<0.00001	81% (72%-90%)	71% (61%-83%)	68% (57%-80%)	7.24 (3.8-13.76)	<0.00001
RCB-III	21 (4%)	60% (42%-86%)	60% (42%-86%)	60% (42%-86%)	9.54 (4.16-21.9)	<0.00001	65% (47%-90%)	65% (47%-90%)	65% (47%-90%)	10.43 (4.24-25.65)	<0.00001
**HR+HER2+ (858)**											
RCB (continuous index)					1.71 (1.51-1.94)	<0.00001				1.87 (1.63-2.15)	<0.00001
RCB Class											
RCB-0	313 (36%)	95% (92%-98%)	93% (90%-96%)	91% (86%-96%)	Ref		96% (94%-99%)	95% (93%-98%)	95% (92%-98%)	Ref	
RCB-I	172 (20%)	96% (92%-99%)	90% (85%-96%)	78% (68%-88%)	2.02 (1.11-3.69)	0.022	98% (95%-100%)	95% (91%-99%)	79% (70%-90%)	2.53 (1.25-5.13)	0.01
RCB-II	291 (34%)	87% (83%-91%)	76% (71%-82%)	64% (57%-72%)	4.12 (2.52-6.76)	<0.00001	90% (86%-93%)	79% (74%-84%)	67% (60%-74%)	5.67 (3.13-10.26)	<0.00001
RCB-III	82 (10%)	72% (63%-83%)	53% (42%-67%)	45% (32%-63%)	8.73 (5-15.22)	<0.00001	73% (64%-84%)	56% (45%-70%)	45% (32%-63%)	12.92 (6.78-24.64)	<0.00001
**HR+HER2+ (Received Neoadjuvant HER2-targed therapy) (756)**											
RCB (continuous index)					1.74 (1.51-2)	<0.00001				1.91 (1.64-2.24)	<0.00001
RCB Class											
RCB-0	290 (38%)	96% (93%-98%)	94% (91%-97%)	91% (86%-97%)	Ref		97% (95%-99%)	96% (94%-99%)	96% (93%-98%)	Ref	
RCB-I	153 (20%)	95% (91%-99%)	91% (85%-96%)	83% (75%-92%)	2.01 (1.01-3.98)	0.046	97% (95%-100%)	95% (91%-99%)	86% (78%-94%)	2.42 (1.06-5.52)	0.036

RCB-II	250 (33%)	88% (84%-92%)	76% (70%-82%)	64% (56%-73%)	4.64 (2.67-8.06)	<0.00001	90% (87%-94%)	79% (73%-85%)	67% (59%-76%)	6.43 (3.27-12.64)	<0.00001
RCB-III	63 (8%)	73% (62%-86%)	54% (40%-71%)	45% (28%-70%)	10.34 (5.42-19.74)	<0.00001	75% (64%-87%)	58% (45%-75%)	44% (27%-71%)	16.2 (7.61-34.5)	<0.00001
**HR+HER2- (1957)**											
RCB (continuous index)					1.55 (1.41-1.71)	<0.00001				1.55 (1.4-1.72)	<0.00001
RCB Class											
RCB-0	217 (11%)	92% (88%-96%)	88% (83%-93%)	81% (73%-91%)	Ref		92% (88%-96%)	89% (84%-94%)	82% (74%-92%)	Ref	
RCB-I	211 (11%)	95% (92%-98%)	91% (86%-95%)	86% (80%-93%)	**0.97 (0.57-1.65)**	0.9	96% (93%-98%)	92% (88%-96%)	87% (81%-94%)	**0.95 (0.55-1.66)**	0.86
RCB-II	1036 (53%)	89% (87%-91%)	80% (78%-83%)	69% (66%-73%)	1.85 (1.23-2.77)	0.0032	89% (87%-91%)	81% (79%-84%)	70% (67%-74%)	1.86 (1.22-2.84)	0.0041
RCB-III	493 (25%)	80% (76%-83%)	71% (67%-76%)	52% (46%-59%)	3.37 (2.22-5.11)	<0.00001	81% (78%-85%)	73% (69%-77%)	53% (47%-60%)	3.38 (2.2-5.21)	<0.00001

*Dark red highlights comparisons where significance was *not* reached (p>0.05)*

Table S3. Multivariate mixed effect Cox models of EFS as a function of RCB as a continuous index, adjusting for age, pre-treatment T category, pre-treatment N status and grade (as fixed effects). Hazard ratios and their 95% confidence intervals with the Wald test p values are shown. †indicates p<0.05

**Variable**	**ALL (4607)**		**HR-HER2- (1585)**		**HR-HER2+ (522)**		**HR-HER2+ (Neoadjuvant HER2-targeted)* (440)**		**HR+HER2+ (773)**		**HR+HER2+ (Neoadjuvant HER2-targeted)* (674)**		**HR+HER2- (1727)**	
	HR	p	HR	p	HR	p	HR	p	HR	p	HR	p	HR	p
**RCB**	1.69† (1.55-1.85)	<0.0001	1.93† (1.74-2.13)	<0.0001	2.09† (1.73-2.53)	<0.0001	2.10† (1.68-2.62)	<0.0001	1.66† (1.45-1.9)	<0.0001	1.69† (1.45-1.97)	<0.0001	1.52† (1.36-1.69)	<0.0001
**Age**	1.00 (0.99-1.00)	0.27	0.99† (0.98-1.00)	0.019	1.00 (0.98-1.02)	0.94	1.00 (0.97-1.03)	0.96	1.00 (0.99-1.02)	0.67	1.00 (0.98-1.02)	0.90	1.00 (0.99-1.01)	0.83
**cT category (Reference: cT2)‡**														
**T0/1**	1.08 (0.85-1.37)	0.53	1.05 (0.69-1.60)	0.81	1.99 (1.00-3.96)	0.051	2.46† (1.03-5.87)	0.042	0.80 (0.4-1.61)	0.53	0.50 (0.2-1.26)	0.14	1.01 (0.69-1.46)	0.97
**T3**	1.28† (1.1-1.49)	0.0012	1.73† (1.37-2.18)	<0.0001	1.60 (0.95-2.69)	0.079	1.64 (0.83-3.24)	0.16	1.02 (0.66-1.56)	0.94	0.88 (0.53-1.48)	0.63	1.08 (0.85-1.37)	0.54
**T4**	1.89† (1.55-2.31)	<0.0001	1.43† (1.02-2.01)	0.036	1.27 (0.6-2.68)	0.53	2.39† (1.02-5.58)	0.044	3.23† (2.07-5.03)	<0.0001	2.98† (1.81-4.9)	<0.0001	2.11† (1.53-2.91)	<0.0001
**cN status (Reference: N-)**														
**N+**	1.15† (1-1.32)	0.047	1.17 (0.94-1.44)	0.15	0.87 (0.52-1.45)	0.59	0.72 (0.38-1.35)	0.30	1.25 (0.84-1.86)	0.27	1.34 (0.85-2.11)	0.20	1.3† (1.04-1.62)	0.023
**Grade (Reference: Grade I/II)**														
**Grade III**	1.51† (1.33-1.72)	<0.0001	1.09 (0.85-1.40)	0.51	0.96 (0.58-1.59)	0.87	0.86 (0.46-1.63)	0.65	0.76 (0.55-1.06)	0.11	0.68† (0.46-0.99)	0.046	1.55† (1.27-1.89)	<0.0001

** The subset who received neoadjuvant HER2-targeted therapy as neoadjuvant treatment in combination with chemotherapy*

‡cT2 was used as the reference category due to the small sample size of the cT0/1 group (particularly within the HER2+ subtypes) out of concern for the stability of the hazard ratio estimates.

Table S4. Multivariate mixed effect Cox models of DRFS as a function of RCB as a continuous index, adjusting for age, pre-treatment T category, pre-treatment N status and grade (as fixed effects). Hazard ratios and their 95% confidence intervals with the Wald test p values are shown. †indicates p<0.05

Variable	ALL (4607)		HR-HER2- (1585)		HR-HER2+ (522)		HR-HER2+ (Neoadjuvant HER2-targeted) (440)		HR+HER2+ (773)		HR+HER2+ (Neoadjuvant HER2-targeted) (674)		HR+HER2- (1727)	
	HR (95% CI)	p	HR (95% CI)	p	HR (95% CI)	p	HR (95% CI)	p	HR (95% CI)	p	HR (95% CI)	p	HR (95% CI)	p
**RCB**	1.75† (1.6-1.9)	<0.0001	1.96† (1.76-2.17)	<0.0001	1.95† (1.62-2.35)	<0.0001	2.08† (1.64-2.63)	<0.0001	1.79† (1.54-2.09)	<0.0001	1.84† (1.55-2.17)	<0.0001	1.50† (1.33-1.7)	<0.0001
**Patient age**	1.00 (0.99-1.00)	0.25	0.99† (0.98-1.00)	0.034	1.00 (0.97-1.02)	0.81	1.00 (0.97-1.03)	0.98	1.00 (0.99-1.02)	0.57	1.00 (0.98-1.02)	0.95	1.00 (0.99-1.01)	0.83
**cT category (Reference: cT2)‡**														
**T0/1**	1.14 (0.89-1.46)	0.31	1.07 (0.69-1.67)	0.75	2.12 (0.98-4.6)	0.057	2.54 (0.97-6.63)	0.056	1.03 (0.51-2.1)	0.94	0.68 (0.27-1.73)	0.42	1.02 (0.7-1.49)	0.92
**T3**	1.34† (1.14-1.56)	0.0003	1.7† (1.34-2.16)	<0.0001	1.99† (1.1-3.59)	0.022	2.09 (0.99-4.43)	0.053	1.19 (0.76-1.86)	0.45	1.09 (0.63-1.89)	0.75	1.10 (0.86-1.41)	0.44
**T4**	1.98† (1.61-2.43)	<0.0001	1.45† (1.02-2.04)	0.038	1.82 (0.85-3.92)	0.13	3.11† (1.27-7.64)	0.013	3.43† (2.12-5.53)	<0.0001	3.63† (2.15-6.12)	<0.0001	2.29† (1.66-3.15)	<0.0001
**cN status (Reference: N-)**														
**N+**	1.14 (0.99-1.32)	0.065	1.18 (0.95-1.47)	0.13	1.00 (0.56-1.79)	0.99	0.69 (0.34-1.4)	0.31	1.24 (0.81-1.89)	0.32	1.37 (0.84-2.23)	0.21	1.31† (1.04-1.65)	0.022
**Grade (Reference: Grade I/II)**														
**Grade III**	1.55† (1.35-1.77)	<0.0001	1.10 (0.85-1.43)	0.45	1.14 (0.64-2.01)	0.65	0.91 (0.45-1.85)	0.80	0.73 (0.51-1.04)	0.085	0.64† (0.42-0.97)	0.036	1.57† (1.28-1.93)	<0.0001

** The subset who received neoadjuvant HER2-targeted therapy as neoadjuvant treatment in combination with chemotherapy*

‡cT2 was used as the reference category due to the small sample size of the cT0/1 group (particularly within the HER2+ subtypes) out of concern for the stability of the hazard ratio estimates.

Figure S1. Prognostic value of RCB overall and by cohort

A-B) Forest plot showing hazard ratio associated with 1 unit increase in RCB from a subtype-stratified fixed effect model for each participating institution for (A) EFS and (B) for DRFS.

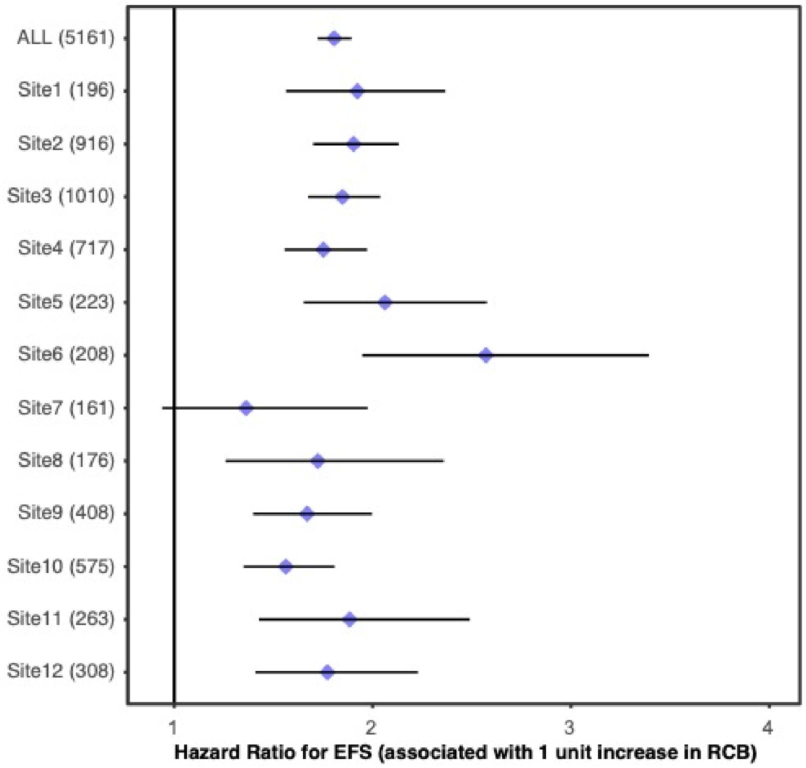

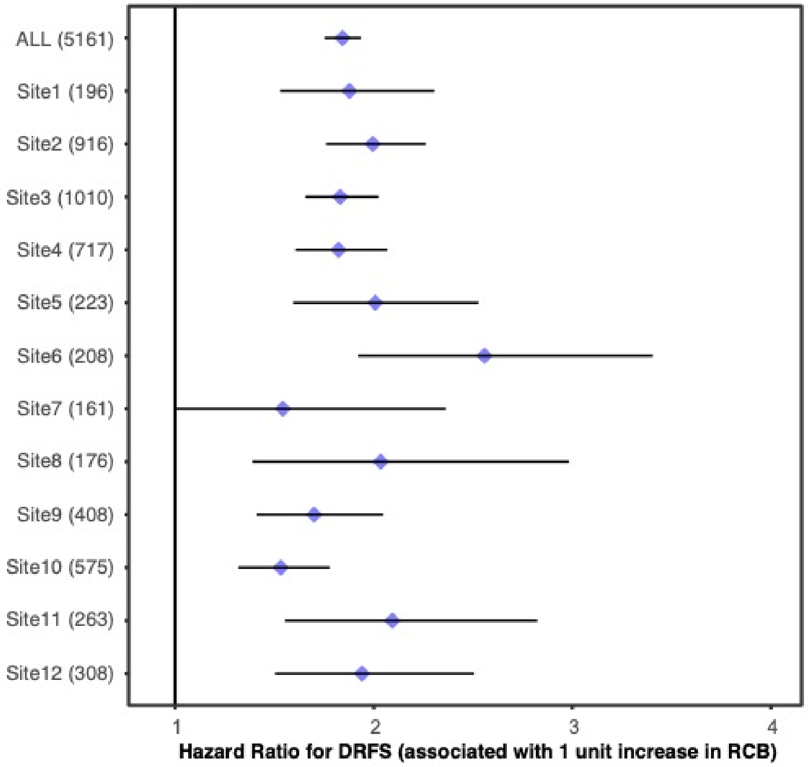

Figure S2. Prognostic value of RCB within HR/HER2 defined subtypes.

A-D) Plot of log relative hazard rate for DRFS events as a function of continuous RCB among (A) HR-HER2-, (B) neoadjuvant HER2 targeted therapy treated HR-HER2+; (C) neoadjuvant HER2 targeted therapy treated HR+HER2+, and (D) HR+HER2- patients. All patients received neoadjuvant chemotherapy. Splines approximation of RCB with 2 degrees of freedom was used to allow non-linear effect.

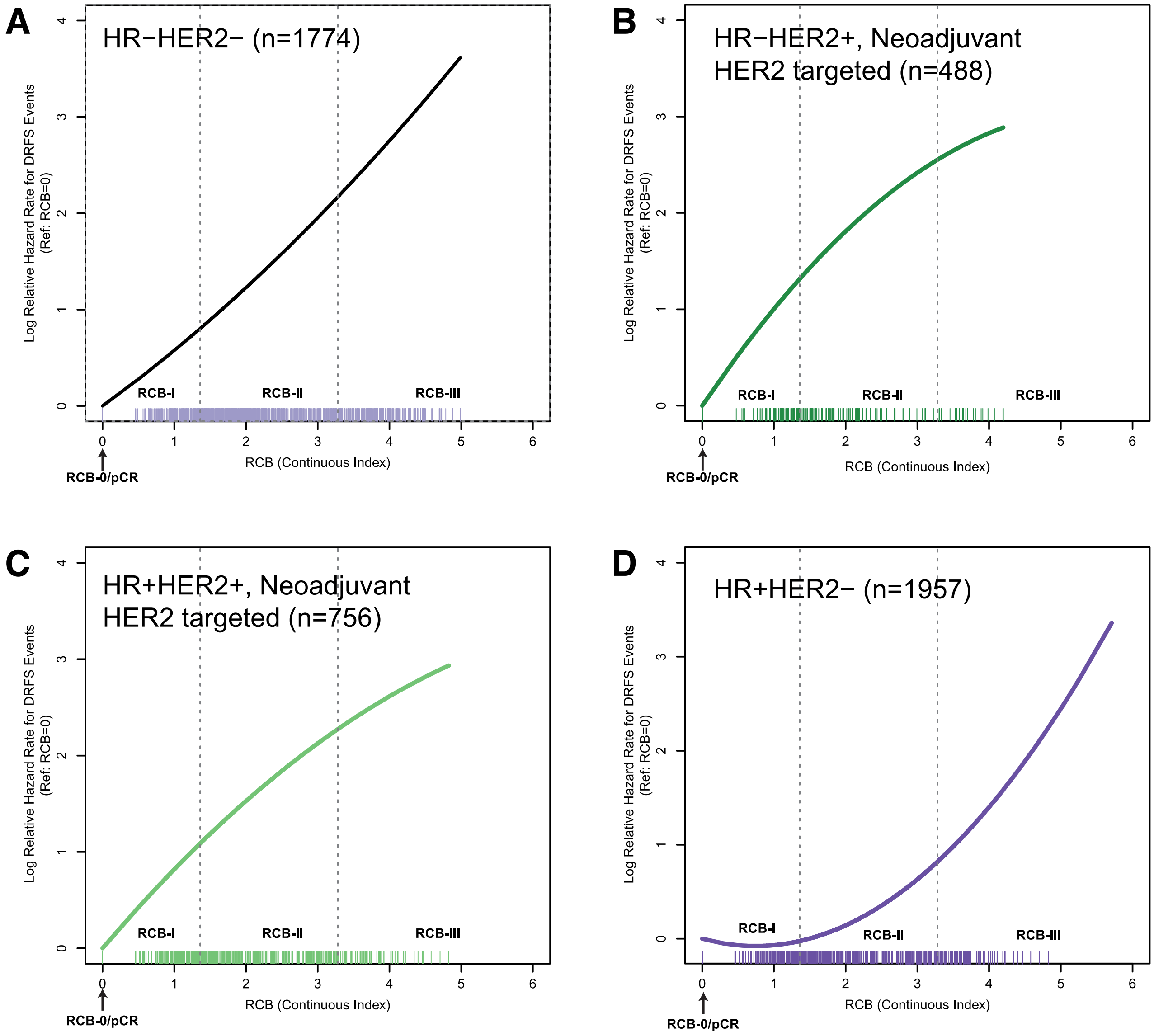

Figure S3. Prognostic value of RCB within HER2-positive subtypes.

(A-B) Plot of log relative hazard rate for EFS as a function of continuous RCB among (A) all HR-HER2+; (B) all HR+HER2 patients. (C-D) Plot of log relative hazard rate for DRFS as a function of continuous RCB among (C) all HR-HER2+; (D) all HR+HER2 patients. All patients received neoadjuvant chemotherapy. Splines approximation of RCB with 2 degrees of freedom was used to allow non-linear effect.

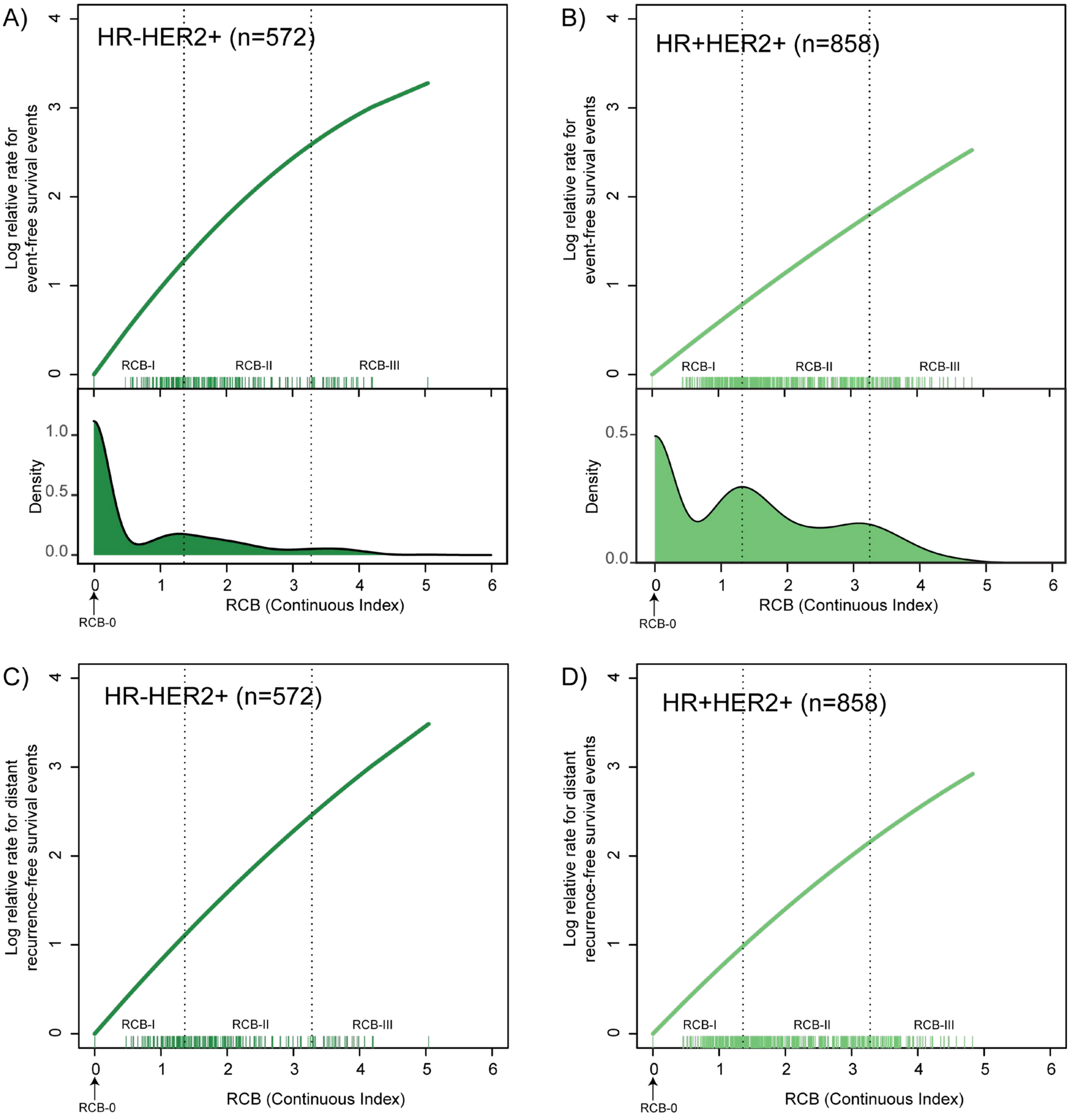

Figure S4. Prognostic value of RCB classes within HR/HER2 defined subtypes.

A-D) KM plots of DRFS by RCB classes for (A) HR-HER2-, (B) neoadjuvant HER2 targeted therapy treated HR-HER2+, (C) neoadjuvant HER2 targeted therapy treated HR+HER2+ and (D) HR+HER2- patients. All patients received neoadjuvant chemotherapy.

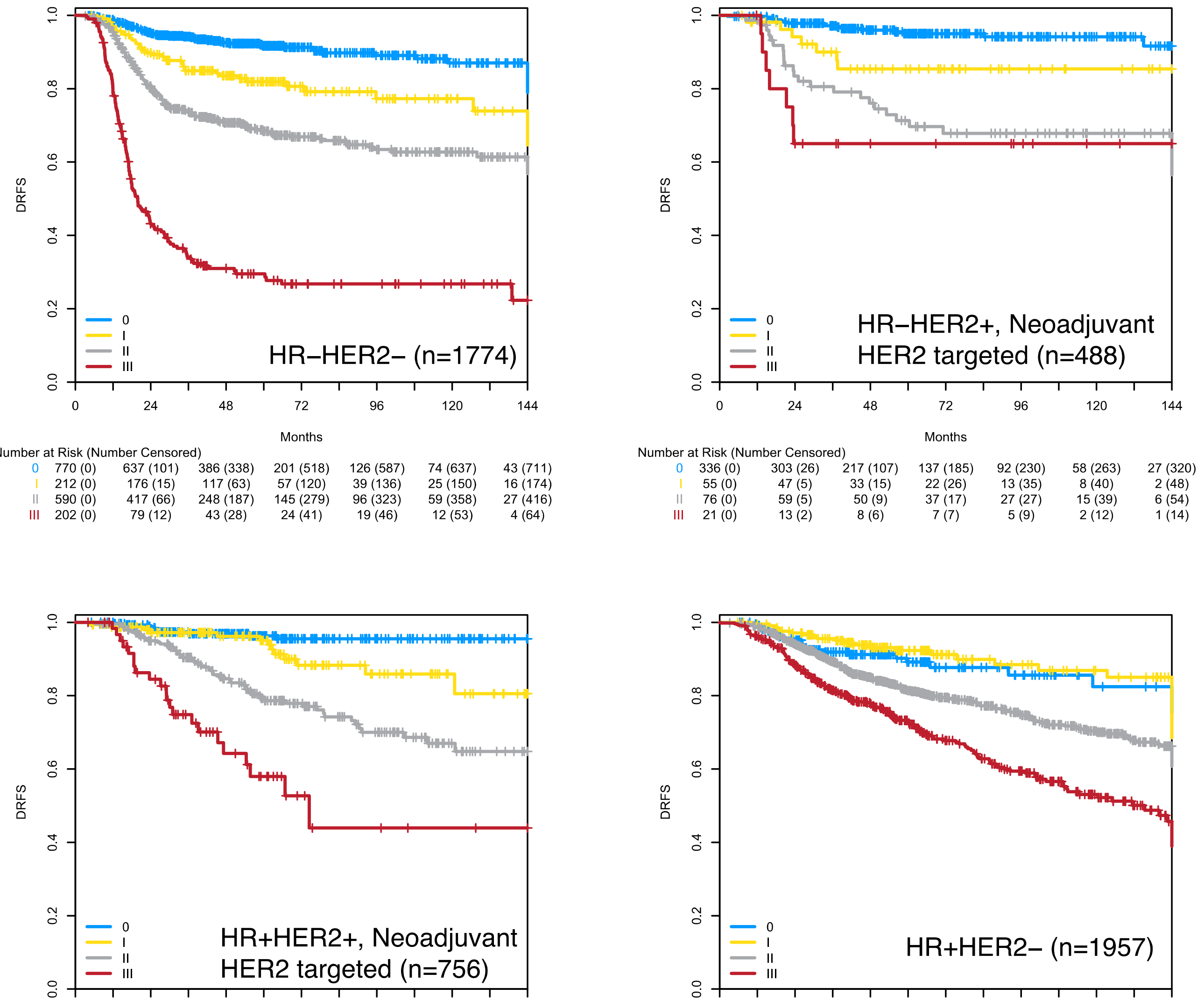

(A) Number at Risk (Number Censored)

	0	24	48	72	96	120	144
0	770 (0)	637 (101)	386 (338)	201 (518)	126 (587)	74 (637)	43 (711)
I	212 (0)	176 (15)	117 (63)	57 (120)	39 (136)	25 (150)	16 (174)
II	590 (0)	417 (66)	248 (187)	145 (279)	96 (323)	59 (358)	27 (416)
III	202 (0)	79 (12)	43 (28)	24 (41)	19 (46)	12 (53)	4 (64)

(B) Number at Risk (Number Censored)

	0	24	48	72	96	120	144
0	336 (0)	303 (26)	217 (107)	137 (185)	92 (230)	58 (263)	27 (320)
I	55 (0)	47 (5)	33 (15)	22 (26)	13 (35)	8 (40)	2 (48)
II	76 (0)	59 (5)	50 (9)	37 (17)	27 (27)	15 (39)	6 (54)
III	21 (0)	13 (2)	8 (6)	7 (7)	5 (9)	2 (12)	1 (14)

(C) Number at Risk (Number Censored)

	0	24	48	72	96	120	144
0	290 (0)	257 (30)	185 (97)	92 (188)	58 (222)	36 (244)	12 (280)
I	153 (0)	130 (19)	93 (55)	53 (89)	33 (108)	16 (125)	4 (140)
II	250 (0)	217 (21)	152 (65)	95 (111)	63 (135)	30 (166)	12 (195)
III	63 (0)	47 (7)	22 (23)	7 (35)	4 (37)	2 (39)	1 (41)

(D) Number at Risk

	0	24	48	72	96	120	144
0	217 (0)	187 (20)	118 (82)	56 (142)	39 (157)	26 (169)	23 (195)
I	211 (0)	197 (8)	135 (64)	74 (122)	59 (135)	47 (146)	36 (192)
II	1036 (0)	921 (55)	613 (284)	377 (486)	279 (565)	188 (641)	109 (820)
III	493 (0)	409 (28)	264 (130)	155 (210)	113 (234)	62 (275)	27 (331)

Figure S5. Prognostic value of RCB class within HER2-positive subtypes.

A-B) KM plots of EFS by RCB classes for (A) all HR-HER2+; (B) all HR+HER2 patients. C-D) KM plots of DRFS by RCB classes for (C) all HR-HER2+; (D) all HR+HER2 patients. All patients received neoadjuvant chemotherapy.

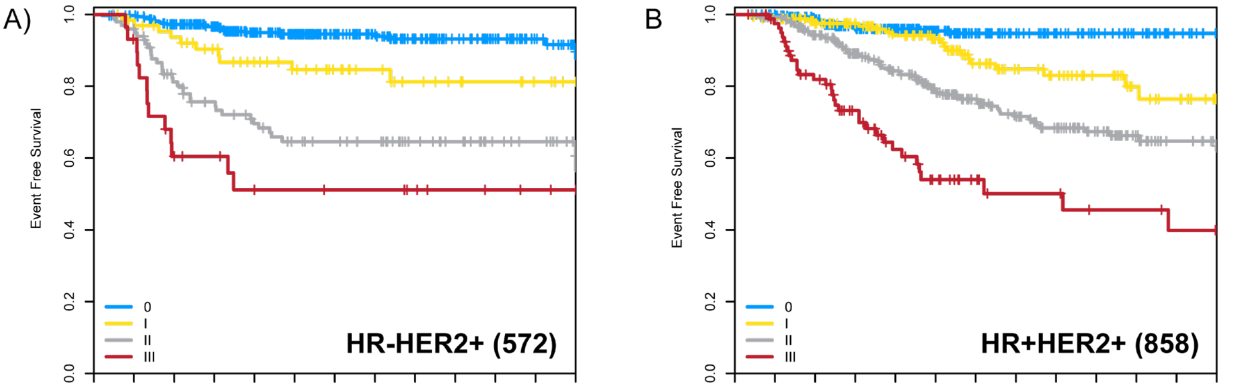

A) Number at Risk

0	376 (0)	339 (27)	248 (108)	167 (187)	118 (235)	81 (270)	47 (303)
I	67 (0)	58 (5)	44 (15)	31 (27)	21 (36)	13 (44)	6 (51)
II	100 (0)	74 (5)	56 (9)	46 (15)	32 (28)	18 (41)	8 (50)
III	29 (0)	16 (2)	10 (5)	9 (6)	7 (8)	4 (11)	2 (13)

B) Number at Risk

0	313 (0)	276 (31)	203 (94)	107 (187)	71 (223)	46 (247)	23 (270)
I	172 (0)	148 (18)	106 (55)	65 (88)	44 (107)	23 (127)	10 (139)
II	291 (0)	251 (22)	180 (65)	118(111)	81 (137)	44 (172)	24 (191)
III	82 (0)	60 (7)	31 (24)	16 (35)	12 (38)	9 (40)	6 (42)

C) Number at Risk

0	376 (0)	339 (27)	252 (108)	170 (188)	119 (238)	82 (274)	48 (307)
I	67 (0)	58 (5)	44 (15)	31 (27)	21 (36)	13 (44)	6 (51)
II	100 (0)	77 (5)	65 (9)	51 (17)	38 (30)	23 (45)	13 (55)
III	29 (0)	18 (2)	10 (6)	9 (7)	7 (9)	4 (12)	2 (14)

D) Number at Risk

0	313 (0)	279 (31)	204 (98)	109 (191)	72 (228)	47 (253)	23 (277)
I	172 (0)	149 (19)	109 (57)	65 (92)	44 (111)	23 (131)	10 (143)
II	291 (0)	253 (22)	184 (67)	122 (114)	83 (142)	45 (178)	24 (198)
III	82 (0)	61 (7)	33 (24)	16 (36)	12 (39)	9 (41)	6 (43)

Table S5. Characteristics of EFS events among hormone-receptor positive HER2 negative RCB-0 patients.

EFS Event	Cohort/ Trial	Patient ER Status	ER-low (1-10% staining or Allred 1-2)	Patient PR.status	PR-low (1-10% staining or Allred 1-2)	Locoregional recurrence (1:Event, 0: No Event)	Distant recurrence (1: Event, 0: No Event)	Death (1: Event, 0: No Event)	Time to EFS Event (Years)
1	7	Positive	Unknown	Unknown	Unknown	0	1	1	0.86
2	9	Positive	No	Unknown	Unknown	1	1	1	0.96
3†	9	Negative		Positive	Yes	0	1	0	1.05
4†	2	Negative		Positive	Yes	1	1	1	1.07
5†	9	Positive	Yes	Negative		0	1	1	1.27
6*	10	Positive	Unknown	Unknown	Unknown	0	1	1	1.38
7†	9	Positive	Yes	Negative		0	1	1	1.53
8†	12	Positive	Yes	Negative		1	1	0	1.55
9*	10	Positive	Unknown	Unknown	Unknown	0	1	1	1.72
10*	10	Positive	Unknown	Unknown	Unknown	0	1	1	1.94
11†	2	Positive	Yes	Negative		0	0	1	2.18
12	3	Negative		Positive	No	0	1	1	2.19
13	9	Positive	No	Unknown	Unknown	0	1	1	2.20
14*	10	Positive	Unknown	Unknown	Unknown	0	1	1	2.23
15*	10	Positive	Unknown	Unknown	Unknown	0	1	1	2.33
16	3	Positive	No	Negative		0	1	1	2.78
17	12	Positive	Yes	Positive	Yes	1	0	0	3.01
18*	10	Positive	Unknown	Unknown	Unknown	1	0	0	3.43
19	3	Positive	No	Negative		0	1	1	3.44
20	3	Positive	No	Positive	No	0	1	0	4.69
21	4	Positive	No	Positive	No	0	1	1	4.96
22	4	Positive	No	Positive	No	0	1	0	5.58
23	4	Positive	No	Positive	Yes	0	1	1	7.66
24	3	Negative		Positive	No	0	0	1	9.90
25†	3	Negative		Positive	Yes	0	0	1	12.27
26	3	Negative		Positive	No	0	0	1	12.28

* Event observed in Artemis trial patient ; † Event observed in ER-negative/PR-low or ER-low/PR-negative patient

Table S6. List of contacts for trials/cohorts within the pooled analysis

Cohort/Trial	Contact	Email
I-SPY1 Trial	Laura Esserman	Laura.Esserman@ucsf.edu
I-SPY 2 Trial	I-SPY 2 Trial data access and publication committee	ispy2dapc@quantumleaphealth.org
MDACC	W Fraser Symmans	fsymmans@mdanderson.org
Institut Curie	Fabien Reyal	fabien.reyal@curie.fr
IISGM	Miguel Martín	mmartin@geicam.org
KUMC	Priyanka Sharma	psharma2@kumc.edu
University of Cambridge (TransNEO and Artemis Trial)	Jean Abraham	ja344@medschl.cam.ac.uk
Yale	Lajos Pustzai	Lajos.Pusztai@yale.edu
Edinburgh	David Cameron	d.cameron@ed.ac.uk
NKI	Gabe Sonke	g.sonke@nki.nl
Mayo	Judy Boughey	boughey.judy@mayo.edu
